# Human Milk Oligosaccharides Inhibit Group B Streptococcal Growth by Binding PcsB, an Essential Cell Wall Separation Protein

**DOI:** 10.1021/jacsau.5c01741

**Published:** 2026-03-27

**Authors:** Julie A. Talbert, Thomas L. Kalmer, Lee S. Cantrell, Mithila D. Bandara, Alexei V. Demchenko, Allison S. Walker, Jennifer A. Gaddy, Steven D. Townsend

**Affiliations:** † Department of Chemistry, 5718 Vanderbilt University, Nashville, Tennessee 37240, United States; ‡ Department of Biochemistry, Vanderbilt University, Nashville, Tennessee 37235, United States; § Department of Chemistry, 7547 Saint Louis University, St. Louis, Missouri 63103, United States; ∥ Department of Biological Sciences, Vanderbilt University, Nashville, Tennessee 37235, United States; ⊥ Department of Medicine, 12328 Vanderbilt University Medical Center, Nashville, Tennessee 37232, United States; # Department of Pathology, Microbiology and Immunology, Vanderbilt University Medical Center, Nashville, Tennessee 37232, United States; ∇ Department of Veterans Affairs, Tennessee Valley Healthcare Systems, Nashville, Tennessee 37212, United States

**Keywords:** group B *Streptococcus*, PcsB, cell wall separation, microscale thermophoresis, proteomics

## Abstract

Human milk oligosaccharides (HMOs) are complex sugars in breast milk that protect babies by preventing harmful bacteria from colonizing the gut. Our team extended the study of HMOs beyond the neonatal gut and characterized their antimicrobial activity against group B *Streptococcus* (GBS), a diplococcus responsible for invasive perinatal infection. To date, the mechanism of action of this antimicrobial activity has remained obscure. To address this key gap, we employed untargeted proteomics, which revealed downregulation of PcsB, an essential murein hydrolase required for cell division. Following successful purification of the active domain of PcsB, we found that this protein domain restores GBS growth in the presence of HMOs, thereby validating PcsB as an HMO protein-interacting partner. *In silico* docking and molecular dynamics simulations predicted that two fucosylated HMOs, lacto-*N*-fucopentaose I (LNFPI) and lacto-*N*-fucopentaose III (LNFPIII), bind to PcsB. *In silico* predictions were validated using microscale thermophoresis assays, which reported dissociation constants of 5 ± 1 mM for LNFPI and 263 ± 72 μM for LNFPIII. Lastly, to test the hypothesis that HMOs may directly modulate the enzymatic activity of the CHAP domain, we employed a turbidimetric assay with commercial PG as the substrate. This assay provided further evidence that HMOs inhibit CHAP. Together, these data suggest that HMOs inhibit GBS growth by binding PcsB at its catalytic site, disturbing essential cell wall separation and division.

## Graphical abstract

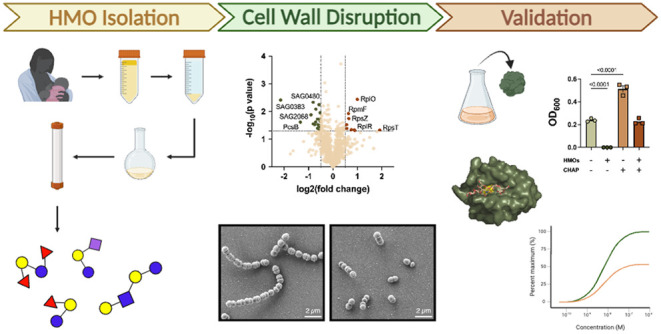

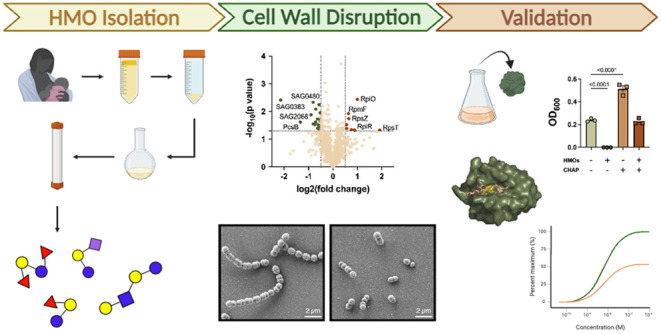

## Introduction

Breastfeeding is widely regarded as the gold standard of infant nutrition, as human breast milk provides complete nourishment for the infant up to six months of life.^1^ Rich in lactose, fats, human milk oligosaccharides (HMOs), proteins, and biomolecules vital for immune function, human breast milk promotes the infant’s health in several ways.^2–5^ Among these components, HMOs serve as prebiotics, enhancing the growth and viability of commensal bacterial species in the microbiome, helping to defend the host against pathogen-associated dysbiosis.^6,7^ HMOs also have direct antagonistic and antiadhesive properties against pathogens. Structurally, over 200 HMOs have been characterized and our previous work has shown that heterogeneous mixtures of HMOs possess antimicrobial activity against a range of bacterial and viral pathogens, including group B *Streptococcus* (GBS).^8–13^ Recently, we demonstrated that this HMO cocktail prevents GBS adhesion to EpiVaginal tissue and gestational membranes, and reduces bacterial burden in a mouse model of ascending vaginal GBS infection during pregnancy.^14^

GBS is a gram-positive bacterium that regularly inhabits the gastrointestinal and urogenital tracts of healthy individuals. Although GBS frequently colonizes healthy individuals without overt disease outcomes, alterations in immune status, such as those present during gestation, can lead to severe invasive GBS infections. Moreover, GBS is a leading cause of neonatal sepsis, stillbirth, pneumonia, and meningitis.^15–18^

Certain structural components are essential to GBS pathogenicity. Rebecca Lancefield initially defined two cell wall-associated carbohydrate antigens in GBS, the capsular polysaccharide (CPS) and the group B-specific carbohydrate (GBC).^19^ The sialic acid-rich CPS, the outermost layer of GBS, shields the cell from phagocytic killing and dampens the host immune response.^20–22^ GBS serotype characterization is based on the specific polysaccharide linkages within the CPS. GBC, which is composed of rhamnose, galactose, *N*-glucosamine, and glucitol, is common to all strains and serotypes of GBS and is positioned on the external cell wall surface.^23^ Both the CPS and GBC are connected to the peptidoglycan (PG) via the *N*-acetylglucosamine (GlcNAc) and *N*-acetylmuramic acid (MurNAc) monomers, respectively.^24–26^ The repeating disaccharide chains of GlcNAc and MurNAc of PG are cross-linked by short peptides consisting of 4–5 amino acids.^27^ Beneath the CPS lies the cell wall and membrane, which are vital to maintaining cell shape and protecting bacterial viability.

As a key component of the cell wall, the mesh-like architecture of PG is critical for cell viability by contributing to morphology, signaling, nutrient transport, virulence, and cell division regulation. As GBS is a *Streptococcus*, it tends to form characteristic chains of cells prior to division. Proper cell division is a highly coordinated process that relies on the assembly of the divisome. This assembly is initiated by FtsZ and its membrane anchor, FtsA, with controlled septal hydrolysis required to separate daughter cells.^28,29^ Disruption to this coordination often results in altered chain morphology and impaired growth.^30–32^

PG hydrolases are essential to facilitate proper cell separation during or after cell division. Recently, the WalKR two-component system (also known as VicKR and YycGF) has been identified as the central regulator of cell wall homeostasis in low G+C Gram-positive bacteria.^33,34^ In *Bacillus subtilis*, *Staphylococcus aureus*, and *Streptococcus pneumoniae*, WalKR regulons contain several PG hydrolases.^35–38^ PcsB, a protein required for cell wall separation in group B *Streptococcus*, is one such PG hydrolase that localizes to the septum to perform hydrolytic function. Isolated full-length PcsB from *S. pneumoniae* or GBS does not exhibit intrinsic hydrolytic activity and, at least in pneumococcus, requires activation by FtsEX.^39–41^ In all bacteria where it has been characterized, including GBS, PcsB is essential for normal growth and cell division.^41–44^

In this report, we employed proteomics to finalize our multiomic approach at characterizing how HMOs inhibit the growth of GBS. Previously published metabolomics and RNA-sequencing (RNA-seq) data sets combined with the proteomic analysis herein revealed a coordinated perturbation of cell division machinery, and most notably, the downregulation of PcsB. Disruption to normal cell wall division was subsequently validated using scanning electron microscopy, illustrating truncated chain length of GBS when the microbe encounters HMOs. Upon purification of the catalytic domain of PcsB, we found that the exogenous protein domain rescued the growth of GBS in the presence of HMOs and that HMOs block the catalytic function of this enzyme. Finally, we employed *in silico* analyses and microscale thermophoresis to identify two binders of PcsB, lacto-*N*-fucopentaose I (LNFPI, *K*_d_ = 5 ± 1 mM) and lacto-*N*-fucopentaose III (LNFPIII, *K*_d_ = 263 ± 72 μM). Together, these data confirm that the interaction of HMOs with PcsB is a prominent factor in their inhibition of GBS growth (Figure 1).

**Figure 1:**
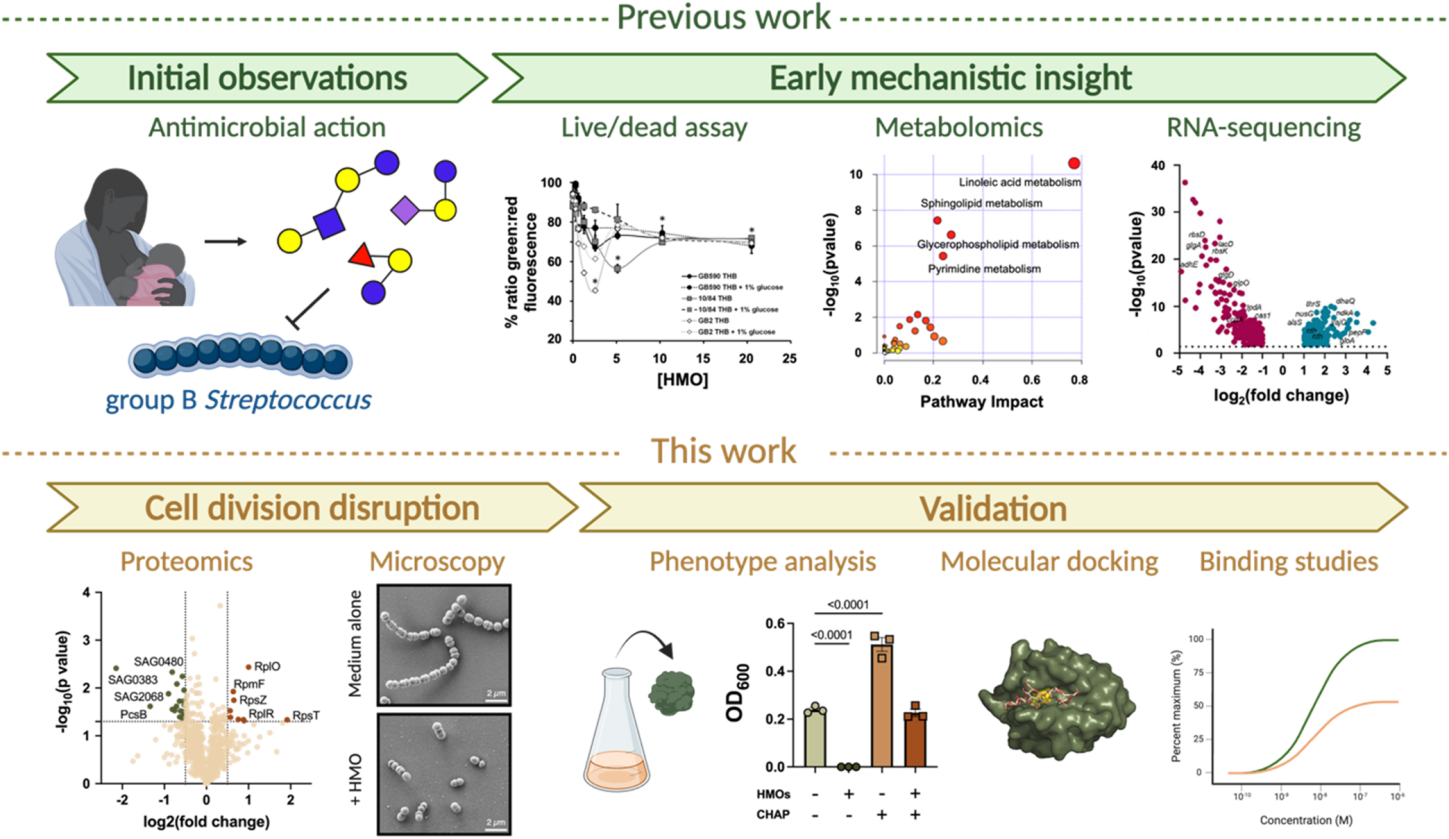
Mechanistic analysis. Initial work demonstrated that HMOs had antimicrobial activity against GBS, potentially due to decreased membrane integrity as visualized via a live/dead BacLight assay. Metabolomics and RNA-seq then identified key disruptions to metabolites and genes with HMO exposure. In this work, we used proteomics and microscopy to identify PcsB as a potential HMO-interacting partner. Then, we demonstrated that the exogenous catalytic domain of PcsB rescues HMO-inhibited GBS growth and that two HMOs, LNFPI and LNFPIII, bind to the active domain.

## Results

### Multiomic Analysis Reveals Disruption to GBS Biochemical Pathways upon HMO Exposure

In previously published work, we employed global untargeted metabolomics using ultrahigh-performance liquid chromatography-high resolution tandem mass spectrometry (UPLC-HRMS/MS) to characterize HMO-mediated perturbation to central metabolism in GBS.^45^ These data showed that multiple metabolic pathways, including linoleic acid (LA) metabolism, sphingolipid metabolism, pyrimidine metabolism, and glycerophospholipid metabolism were significantly perturbed upon exposure of GBS to HMOs (Table S1). In a second critical study, we demonstrated that HMOs reduce GBS adherence *ex vivo* and decrease bacterial burden in a murine model of ascending vaginal GBS infection.^14^ Therein, we introduced data collected from RNA-seq comparisons between GBS in medium alone to medium supplemented with HMOs (Figure S1). With over 400 genes showing significant differences between untreated and treated samples, it is apparent that HMOs disrupted multiple systems at the RNA level. Herein, we evaluated changes in the proteome of GBS upon HMO supplementation (Figure 2). For untargeted proteomic analysis, we compared GBS strain GB00590 (GB590) in unsupplemented medium to medium supplemented with HMOs (2 mg/mL). All HMOs used for omics were isolated from 5–7 donors and pooled to create a heterogeneous mixture. HMO concentrations were selected to inhibit cell proliferation while allowing enough growth for analysis.

**Figure 2:**
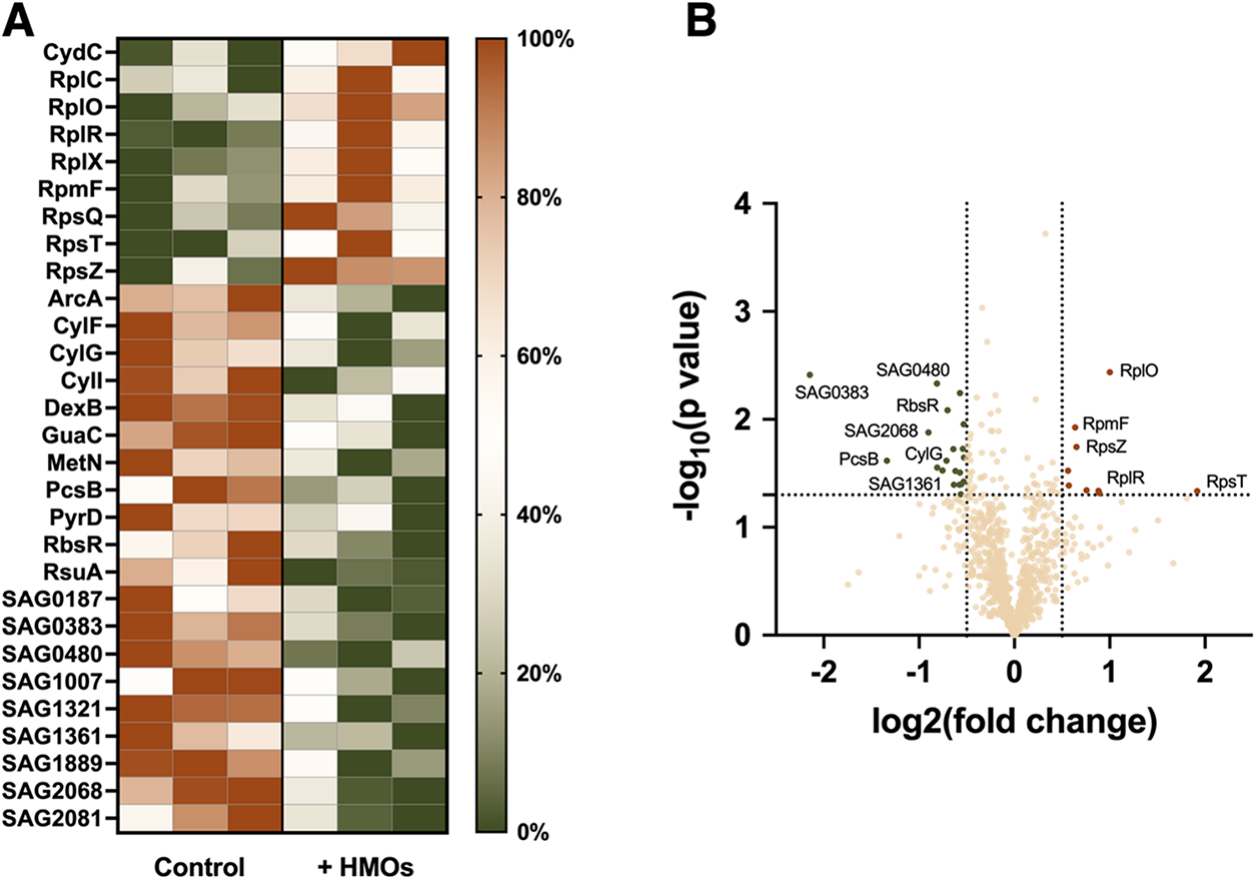
Proteomic analysis reveals changes in protein abundance in GBS with exposure to HMOs. (A) Heat map visualization of all differentially produced proteins by normalized abundance. Colors displayed indicate high abundance (dark orange) to low abundance (dark green). (B) Volcano plot of proteins that exhibited downregulation (dark green) versus proteins that exhibited upregulation (dark orange). Significance was defined as having a log2 fold change of ±0.5 and *p*-value ≤0.05.

Integration of these omics methods strongly suggested that HMOs disrupt the cell wall and membrane (discussed below) as well as nucleotide synthesis. Specifically, all three data sets unveiled perturbations to pyrimidine synthesis. In both the RNA-seq and proteomics, there was decreased expression and downregulation of the pseudouridine synthase gene, *rsuA*, and its protein product, RsuA, respectively. Pseudouridine synthases are responsible for the post-transcriptional modification in which uridines are isomerized. Decreased expression and downregulation was also seen for a ribose operon repressor, which regulates the metabolism of ribose and the *de novo* biosynthesis of purine nucleotides.^46^ Finally, *guaC* encodes a GMP reductase, which catalyzes the deamination of GMP to IMP, a process that is crucial for balancing nucleotide levels.^47^ The gene and protein product experienced decreased expression and downregulation in the RNA-seq and proteomics, respectively. Although nucleotide synthesis was clearly implicated in each omic method, cell wall disruptions represented the most significant finding, which we examine in detail in the next section.

### HMO Exposure Triggers Major Disruptions to Cell Division

Bacterial division is a highly coordinated process that relies on cell wall building with septation and elongation, ensuring proper bacterial growth and division. Peptidoglycan (PG), or murein, is the structural backbone of the cell wall and consists of repeating units of *N*-acetylglucosamine (GlcNAc) and *N*-acetylmuramic acid (MurNAc) monomers. From the metabolomics analysis, one cell wall-affiliated metabolite, UDP-MurNAc was significantly accumulated in the HMO-treated samples (Table 1).^45^ PG biosynthesis was also implicated in the RNA-seq with differential expression of three *mur* genes, *murA*, *murC*, and *murM*. The *murA* gene encodes an enzyme that catalyzes the addition of enolpyruvate group to UDP-GlcNAc to form UDP-MurNAc, while *murC* encodes an enzyme that pushes UDP-MurNAc forward in PG biosynthesis by adding alanine. As *murA* expression was increased at the RNA level while *murC* was decreased, the accumulation of UDP-MurNAc observed in the metabolomics data is consistent with these transcriptional changes. Finally, *murM*, which exhibited decreased expression in the RNA-seq data set, is responsible for attaching l-Lys to Lipid II, the PG precursor, leading to branched side chains.

**Table 1:** Metabolites and Genes Involved in PG Biosynthesis That Were Significantly Changed with HMO Exposure

select metabolites^a^	select genes^a^	*P*-value	fold change
UDP-MurNAc		0.032	19.2
	*murC*	0.001	–1.10
	*murM*	0.0009	–1.09
	*murA*	0.0029	0.92

a Full analysis in previous publications.^14,45^

Cell division is initiated by assembly of the divisome at midcell, with FtsZ polymerization marking the division site. This initiation recruits additional proteins, including FtsA and penicillin binding proteins, to drive septal PG synthesis. This PG synthesis explicitly relies on the precursor, Lipid II, produced via the Mur pathway. In our data sets, *ftsA* and *pbp2b* exhibited increased expression at the RNA level (Table 2).

**Table 2:** Proteins and Genes Involved in Cell Division or Cell Wall Remodeling That Were Significantly Changed with HMO Exposure

select proteins	select genes^a^	*P*-value	fold change
PcsB		0.0242	–1.34
SAG1889		0.0404	–0.57
	*ftsA*	0.0072	0.65
	*ftsE*	0.0027	–0.99
	*ftsX* ^b^	0.0533	–1.11
	*pbp2b*	0.0009	1.23
	*sag1889*	8.57 × 10^–6^	2.99

a Full analysis in previous publications.^14,45^

b not statistically significant.

Critical for proper cell division, cell wall separation is performed by PG hydrolases, such as PcsB (a protein required for cell wall separation in group B *Streptococcus*), which exhibited downregulation in the proteomics data with HMO treatment, but showed no significant difference in transcript abundance (Figure 2 and Table 2).^14^ Reinscheid’s group was the first to characterize PcsB in GBS by finding that the conditional deletion of *pscB*, requiring 500 mM sorbitol for experimental assays, drastically reduced growth rates and increased susceptibility to several antibiotics.^41,42^ Full-length PcsB, which does not have hydrolytic activity in GBS^41^ or *S. pneuominae*,^40^ is hypothesized to be activated by the protein products of *ftsEX*. Notably, *ftsE* exhibited decreased expression in the RNA-seq data. Structurally, FtsEX is an ATP-binding cassette (ABC) family complex widely conserved across bacterial genera and implicated in cell division in *Escherichia coli*,^48,49^ *Mycobacterium tuberculosis*,^50^ and *S. pneumoniae*.^40,51–53^

Notably, the Δ*pcsB* mutant from Reinscheid’s work could not form the characteristic chain-like structures of GBS and, instead, grew in clumps with multiple division septa per singular cell. When investigating the proteomic analysis leads, we leaned on our seminal work on HMOs with GBS, which demonstrated that supplementation of HMOs from a singular donor caused the similar clumping morphological changes as seen with the Δ*pcsB* mutant.^9^ To investigate further, we used focused ion beam scanning electron microscopy (FIB-SEM) with a subinhibitory concentration of a pooled mixture of HMOs from several donors. The micrographs demonstrated that HMOs significantly truncated the chain length of GB590 (*p* < 0.0001, Student’s *t* test; Figure 3A,B), partially mimicking the phenotype seen from our original paper and that of the Δ*pcsB* mutant.

**Figure 3:**
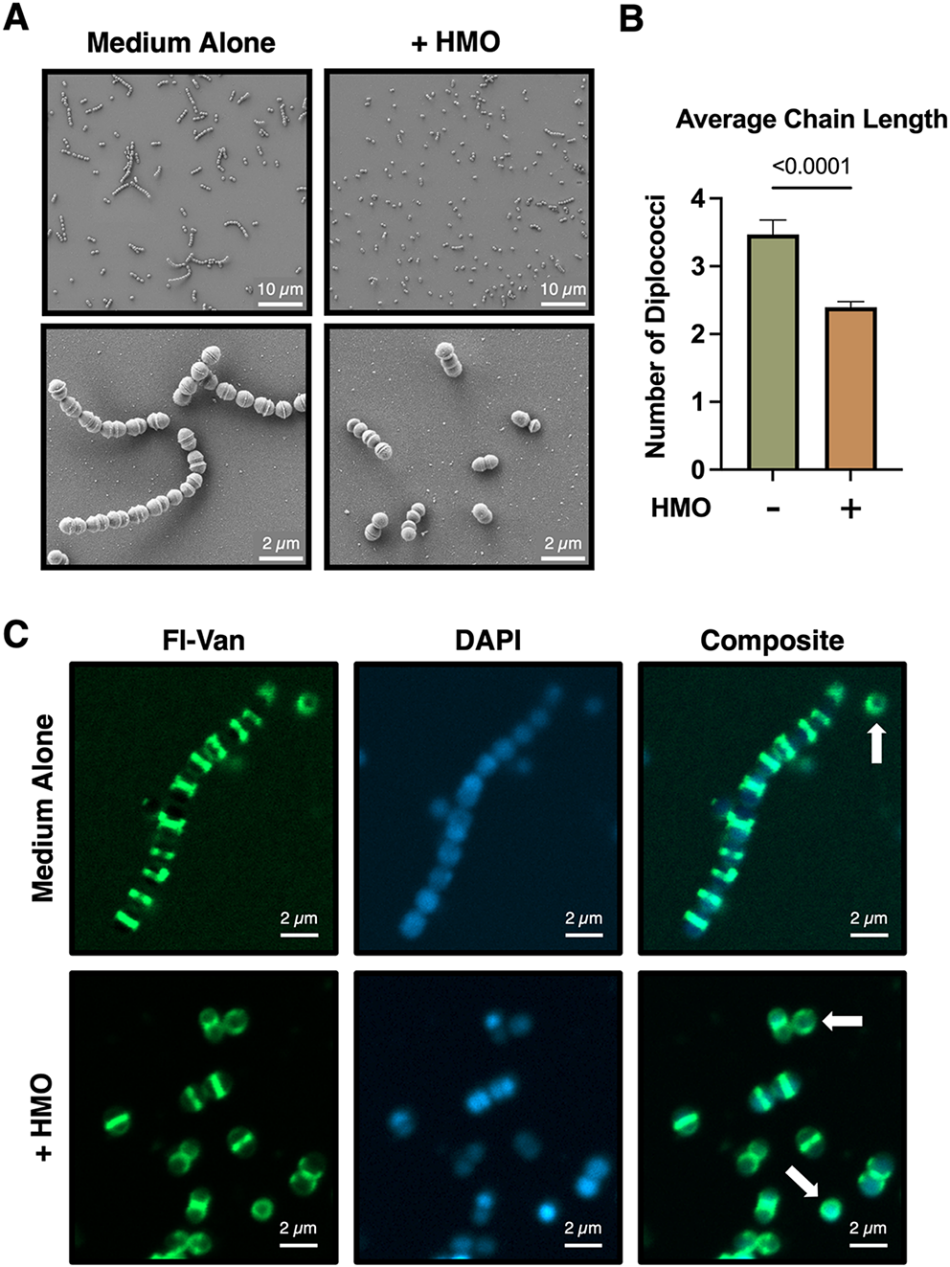
Microscopy analyses indicate that HMOs truncate the chain length of GBS. (A) FIB-SEM analyses of GB590 grown with and without HMOs at a subinhibitory concentration. (B) HMOs significantly decrease the average chain length of GBS as measured by diplococci from scanning electron micrographs in A (*p* < 0.0001, Student’s *t* test). Over 130 chains were counted for each condition. (C) Fluorescent vancomycin (Fl-Van) and DAPI staining to investigate HMO-mediated cell wall synthesis disruption with a subinhibitory concentration of HMOs as shown via CLSM. Micrographs are representative of 3 biological replicates.

To further investigate whether HMOs cause erratic cell wall separation, we used confocal laser scanning microscopy (CLSM). GB590 was stained with DAPI and a fluorescent vancomycin (Fl-Van), which detects active cell wall synthesis regions.^54^ When a subinhibitory concentration of HMOs was added, green parallel bands were observed at most septal regions, indicating proper cell wall synthesis. (Figure 3C). Singular cells in both samples, however, have a diffuse staining pattern due to the lack of septa as indicated by the white arrows in Figures 3C and S2. Since the untreated sample has more chains than that of the HMO-treated, this diffuse staining could solely appear more prevalent in the HMO-treated sample because of an increased number of singular cells. As such, the most considerable observed difference in the HMO-treated vs untreated samples was the decrease in chain formation as seen via SEM.

### Exogenous CHAP Domain Enables GBS Growth in the Presence of HMOs

With microscopy data suggesting PcsB as a potential interaction partner with HMOs, we sought to purify the protein from GBS to investigate its activity *in vitro*. However, PG hydrolases require activation to perform their function, as discussed previously. Håverstain and Hermoso reported on the dimeric crystal structure of pneumococcal PcsB, which traps the carboxy-terminal cysteine, histidine-dependent amidopeptidase (CHAP) catalytic domain of each dimeric partner in an inactive configuration.^40^ This CHAP domain is responsible for the cleavage of the peptide chain in PG, allowing cell wall separation.^41,42^ The opening of the dimeric molecular tweezers likely requires an ATP-driven conformational change in the FtsEX complex to release the catalytic CHAP domain. In this vein, we obtained a pGS-21A vector, containing glutathione-S-transferase (GST) for solubility, harboring the active CHAP domain sequence from GenScript (Piscataway, NJ). Successful purification yielded the final construct of His-GST-His-CHAP (“tagged CHAP”) (Figure S3). GST was included in the protein construct to increase solubility of the domain after several attempts of protein purification without GST led to <60 μg/mL of nonpure protein, which was unusable for our bacterial assays.

The activity of purified tagged CHAP was analyzed via growth curves using a heterogeneous mixture of HMOs that were isolated and pooled from human breast milk.^9,11^ Activity was evaluated by comparing the growth, as measured by spectrophotometric analysis of the optical density at 600 nm (OD_600_), of GBS strain COH1 in medium alone to growth in medium containing protein, HMOs, or both (Figure 4A). COH1 was used as it is a commonly employed representative strain of serotype III GBS strains. COH1 growth was significantly inhibited in the presence of HMOs at their minimum inhibitory concentration (MIC, 15 mg/mL for mixture used for these assays) (*p* = 0.0024 at 24 h via two-way ANOVA with Dunnett’s post hoc test; Figure 4A). Addition of tagged CHAP (10 μM) significantly increased the growth of COH1 via OD_600_ readings (*p* = 0.0140 at 24 h via two-way ANOVA with Dunnett’s post hoc test, Figure 4A). However, because FIB-SEM revealed an increased presence of cell debris, and because 24 h viability data showed no statistically significant difference in GBS growth between medium alone and medium with tagged CHAP, the increased OD_600_ is likely due to increased cell debris (Figure 4B). The micrographs also demonstrate that tagged CHAP caused the streptococcal chains to be longer (*p* < 0.0001 via one-way ANOVA with Tukey’s post hoc test; Figure 4D).

**Figure 4:**
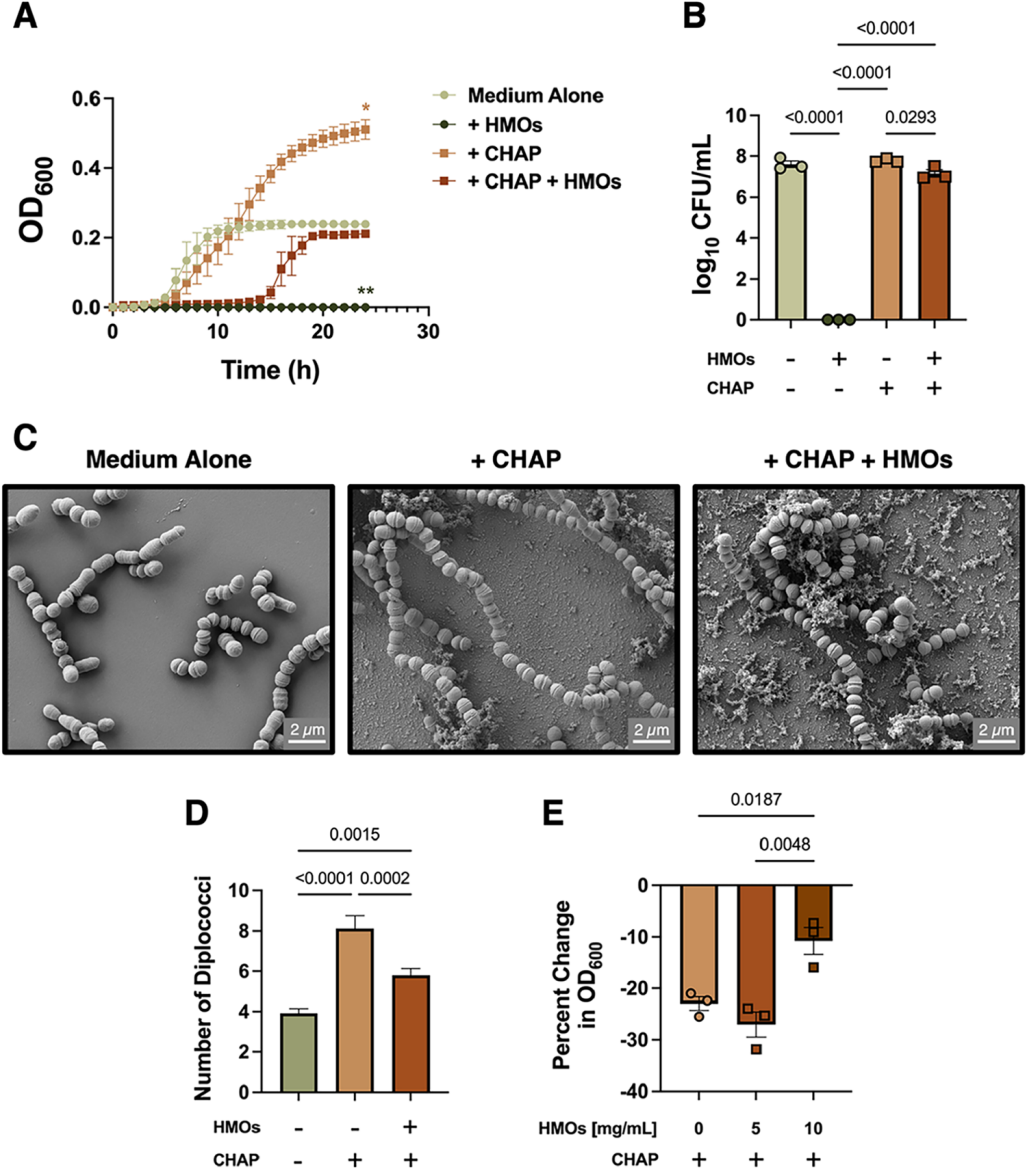
Exogenous CHAP domain rescues the growth of GBS in the presence of HMOs. (A) Growth analyses over 24 h demonstrate that tagged CHAP rescues GBS growth with HMOs present. (B) Viability data collected at 24 h show only decreased log_10_(CFU/mL) with HMO supplementation. Symbols indicate mean ± SEM (C) FIB-SEM analyses of COH1 grown alone or in the presence of tagged CHAP ± HMOs. Micrographs are representative of 3 biological replicates. (D) Tagged CHAP ± significantly increases the average chain length of GBS as measured by diplococci from scanning electron micrographs. Over 130 chains were counted for each condition. (E) HMOs inhibit PG lysis by tagged CHAP in a dose-dependent manner as measured by the percent change in OD_600_. *p* values calculated via two-way ANOVA (panel A, *p = 0.0140, **p = 0.0024) or one-way ANOVA (panel B, D, E, *p* values on graph) with Dunnett’s post hoc test, *N* = 3 for panel A and B.

Gratifyingly, exogenous tagged CHAP domain (10 μM) rescued the growth of COH1 in the presence of HMOs at their MIC (Figure 4A). Micrographs showed a combination of normal and elongated GBS chains, indicating that tagged CHAP overcomes the chain-truncation effect observed with HMOs (Figure 4C). Indeed, HMOs and tagged CHAP create significantly longer chains than medium alone, but shorter than with tagged CHAP alone (*p* = 0.0015 and *p* = 0.0002, respectively, via one-way ANOVA with Tukey’s post hoc test; Figure 4D).

To ensure the solubility tag was not rescuing the growth of COH1 and that the growth rescue was not due to random protein-HMO interactions, we also ran these growth assays with GST (10 μM) alone and in combination with HMOs (Figure S4). GST was unable to rescue the growth of COH1. Notably, while the Reinscheid group made a conditional knockout of *pcsB*, they noted the use of 500 mM sorbitol to stabilize the cell wall long enough for data collection.^41,42^ As *pcsB* is an essential gene for GBS survival, as demonstrated by Dr. Thomas Hooven's and Dr. Adam Ratner's groups,^55^ we opted to purify the protein to study its role in interactions with HMOs.

### HMOs Inhibit PG Cleavage by Tagged CHAP

Given that tagged CHAP rescued COH1 growth in the presence of HMOs, we hypothesized that these oligosaccharides might directly modulate the enzymatic activity of the CHAP domain. To test this, we employed a turbidimetric assay with commercial PG from *S. aureus* as the substrate. Although the pentaglycine cross-bridge of *S. aureus* PG differs from the peptidebridge found in GBS, CHAP domains often exhibit catalytic promiscuity across diverse PG structures. While CHAP_PcsB_ is predicted to function as an *N*-acetyl-muramoyl-l-alanine amidase, cleaving the bond between MurNAc and the l-Ala of the stem peptide,^41^ biochemical confirmation of this specific activity is lacking.

The commercial PG was suspended at 2 mg/mL in assay buffer to create a turbid substrate environment. Tagged CHAP (15 μM) was added to this suspension, resulting in an averaged 22% reduction in the OD_600_. However, the addition of HMOs inhibited this PG cleavage in a dose-dependent manner. While 5 mg/mL HMO did not significantly alter activity, 10 mg/mL significantly reduced the percent change in OD_600_ (*p* = 0.0187 via one-way ANOVA with Tukey’s post hoc test; Figure 4E).

### In Silico Modeling of the Active Site of PcsB

The HMO mixture used in the phenotypic assays is a heterogeneous concoction of HMOs pooled from the breast milk of several donors. To narrow down which single-entity HMOs in this cocktail could be binding to PcsB, we first employed AlphaFold to predict the structure of the CHAP domain for use in molecular docking assays (Figure S5).^41,42,56^ The CHAP domain has similar characteristics to other papain-like hydrolytic enzymes, characterized as cysteine proteases. The catalytic activity of these proteins lies in their formation of covalent intermediates with their substrates. A reactive Cys acts as the catalytic nucleophile, promoted by a nearby His. The His-imidazole ring is hydrogen bonded to a third catalytic residue, normally Asp or Asn.^57,58^ In *S. pneumoniae*, the third residue of the catalytic triad is Glu.^40^ Sequence alignment shows that the three catalytic residues of CHAP_PcsB_ of *S. pneumoniae* (Cys292, His343, Glu360) match an identical three residues in that of GBS strain COH1 (Cys347, His400, Glu417) (Figure S6A). Further, the catalytic triad, consisting of Cys, His, and Asp/Asn/Glu, is usually positioned by the structural core for papain-like proteases. The Cys is often located at the N terminus of a helix, with the β-sheet of four antiparallel strands and linking loops providing the His and Asp/Asn/Glu.^59^ Superimposition of CHAP_PcsB_ of GBS and *S. pneumoniae* shows strong overlap of the catalytic triad with residue locations matching the common structural core of these types of proteins (Figure S6B). Unsurprisingly, there were various structural similarities between CHAP_PcsB_ of GBS and *S. pneumoniae* including the length and width of the active site groove (31 Å and 7 Å, respectively (Figure S6C)).^40^

A pentapeptide commonly found in Gram-positive PG,^27^ l-Ala-D-iGln-l-Lys-d-Ala-d-Ala, was optimized using Avogadro and used to gain insight into substrate recognition by CHAP_PcsB_.^60^ Top scoring positions fit the pentapeptide in the active site of PcsB with the scissile bond placed in proximity to the catalytic Cys.^40,61^ Electrostatic potential on the CHAP_PcsB_ surface indicates that l-Lys is docked preferably to avoid the basic patches (colored in blue, Figure S6D) with stabilization by Glu417 (Figure S6E). Further, various hydrogen bond interactions in the active site stabilize the peptide stem (Figure S6E).

### In Silico Predictions Support HMO–CHAP Binding

With the catalytic triad identified, we moved to model how HMOs could interfere with cell wall separation using AutoDock Vina^62,63^ and the AlphaFold predicted structure of CHAP_PcsB_. As the HMO cocktail is a heterogeneous mixture of oligosaccharides, we used select Avogadro-optimized HMOs for the docking studies relying on previously published data from our group that characterized the antimicrobial activity of 16 HMOs (5 mg/mL) against GB590 (Table 3).^64,65^ The top scoring positions of all active HMOs were predicted to fit the oligosaccharide into the active site groove of CHAP (Figures 5B, S7B, and S8B). Intriguingly, some of top scoring positions of inactive HMOs were predicted to fit the HMO in the active site, with two being predicted to bind elsewhere on the domain (Figures 5B, S7B, and S9B).

**Figure 5:**
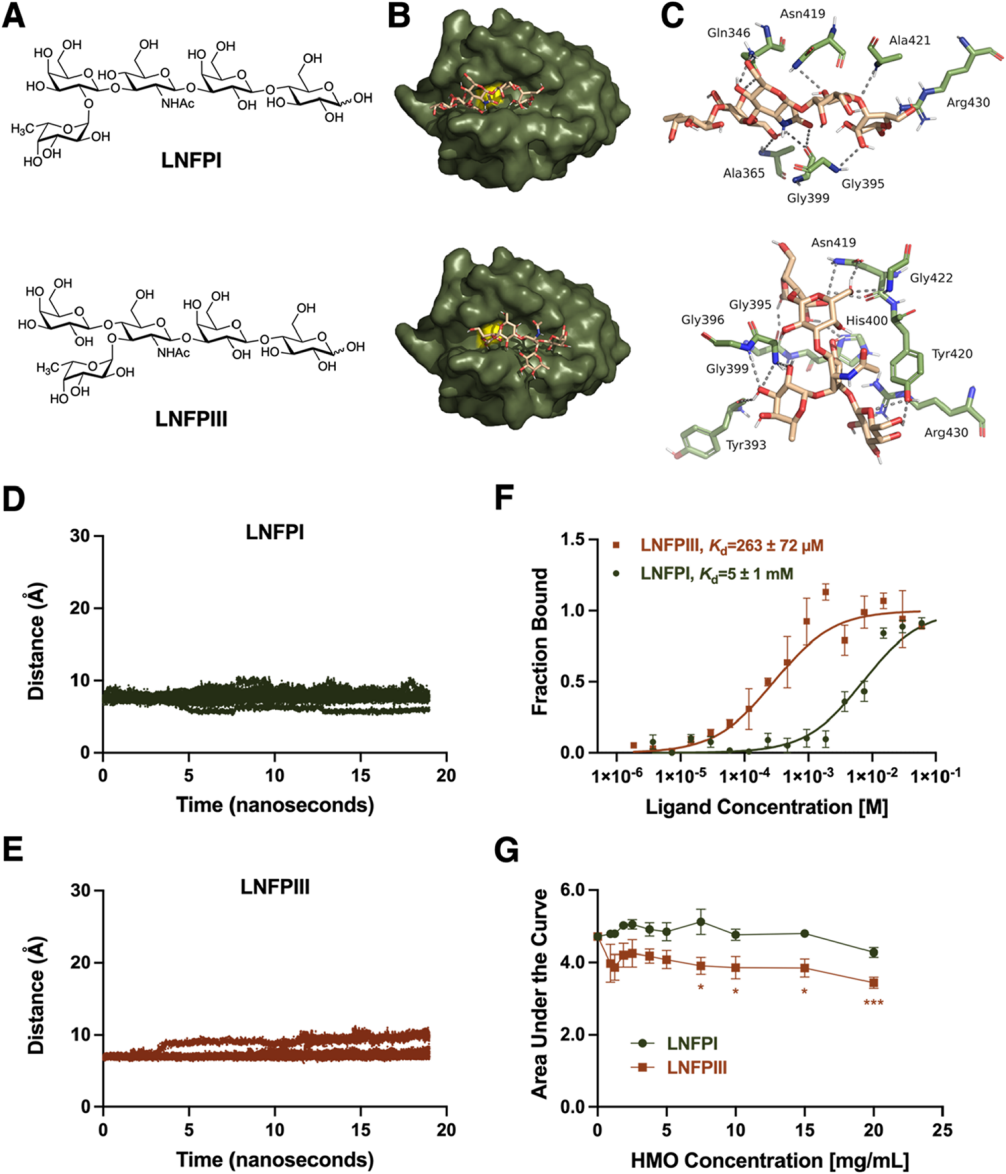
Lacto-*N*-fucopentaose I (LNFPI) and lacto-*N*-fucopentaose III (LNFPIII) bind to CHAP_PcsB_. (A) Structures of LNFPI and LNFPIII. (B) Docking reveals both LNFPI and LNFPIII dock in the active site of PcsB. (C) Interactions of LNFPI and LNFPIII with residues in active site. MD simulation data (10 replicates) demonstrating that LNFPI (D) and LNFPIII (E) are predicted to remain in the active site of PcsB over 19 ns. (F) Microscale thermophoresis reveals dissociation constants of 5 ± 1 mM for LNFPI and 263 ± 72 μM for LNFPIII. (G) LNFPIII (7.5, 10, 15, and 20 mg/mL) significantly reduces the growth of COH1 (*p < 0.03, ***p = 0.003; one-way ANOVA with Dunnett’s post hoc test, *N* = 3), while LNFPI has no significant activity.

**Table 3:** Summary of Results from Molecular Docking Experiments

substrate	antimicrobial activity in GB590^a^	docks in active site	affinity (kcal/mol)^b^	binds His400
human milk oligosaccharides				
2’-fucosyllactose (2’-FL)	no	no	N/A	no
3-fucosyllactose (3-FL)	no	no	N/A	no
*para*-lacto-*N*-neohexaose (*para*-LNnH)	no	yes	–6.9	yes
lacto-*N*-fucopentaose I (LNFPI)	no	yes	–6.6	no
LS-tetrasaccharide c (LST c)	no	yes	–6.4	no
disialyllactose-*N*-tetraose (DSLNT)	no	yes	–6.2	no
3′-sialyllactose (3′-SL)	no	yes	–6.1	no
6′-sialyllactose (6′-SL)	no	yes	–5.8	no
difucosyllactose (DFL)	yes	yes	–7.4	yes
lacto-*N*-neohexaose (LNnH)	yes	yes	–7.2	yes
lacto-*N*-fucopentaose III (LNFPIII)	yes	yes	–6.9	yes
lacto-*N*-triose II (LNTII)	yes	yes	–6.9	yes
LS-tetrasaccharide a (LST a)	yes	yes	–6.7	yes
lacto-*N*-tetraose (LNT)	yes	yes	–6.4	yes
lacto-*N*-fucopentaose II (LNFPII)	yes	yes	–6.3	yes
lacto-*N*-neotetraose (LNnT)	yes	yes	–6.3	no
PG peptide stem				
l-Ala-d-iGln-l-Lys-d-Ala-d-Ala	N/A	yes	–5.8	no

a Measured at 24 h.

b Calculations performed by AutoDock Vina.

Seven of the eight active HMOs were predicted to bind to the side chain of His400 (Figures 5C, S7C, and S8C), the residue involved in the thiolate/imidazolium ion pair. Experimentally, if that critical nitrogen is engaged in a hydrogen bond with the HMO, we hypothesize that Cys347 is unable to function as a nucleophile and attack the carbonyl carbon of the scissile bond. Of the nonantimicrobial HMOs that dock in the active site, only the top scoring position of one, *para*-lacto-*N*-neohexaose (*para*-LNnH), was predicted to engage in a hydrogen bond with His400 (Figures 5C, S7C, and S8C). Additionally, all antimicrobial HMOs have a higher predicted affinity for the active site of PcsB than the modeled PG pentapeptide (Table 3).

### LNFPI and LNFPIII Bind to CHAP_PcsB_

To further assess the protein–ligand interactions described herein, we performed molecular dynamics (MD) simulations of all HMOs that were predicted to dock in the active site groove of PcsB using AMBER. Each HMO was subjected to ten replicates of 19 ns each, unless the HMO reached a certain distance threshold, at which point the run was halted. From these data, LNFPI, LNFPIII, LNnH, and *para*-LNnH remained the closest to the active site of PcsB (Figures 5D,E and S7DE). The remaining 10 HMOs diffused away from the active site (Figure S10).

To validate this *in silico* work, we employed microscale thermophoresis (MST) to evaluate the binding of LNFPI and LNFPIII to a His_6_-tagged CHAP domain purified by GenScript (Piscataway, NJ, Figure S11). To determine the required incubation time, a kinetic MST analysis was performed for LNFPI, monitoring the bound fraction over 150 min. While the raw bound values continued to exhibit small increases, the average fraction bound value was already high (0.990) at the 15 min time point (Table S2). Crucially, the dissociation constant (*K*_d_) determined from the 15 min time point was not statistically different from the *K*_d_ determined at 60, 105, or 150 min (Table S2). Given that binding was already 99.0% complete at 15 min with negligible differences in *K*_d_ values, a 10–15 min incubation was implemented for all MST assays. MST yielded *K*_d_ values of 5 ± 1 mM for LNFPI and 263 ± 72 μM for LNFPIII (Figure 5F). Because protein-carbohydrate interactions have historically been difficult to characterize, we attribute the relatively high standard deviations to their transient nature.

While the vast difference in *K*_d_ matches our previous data, deeming LNFPI as an “inactive HMO” at 5 mg/mL against GB590 (Table 3), we hypothesized that a higher physiologically relevant concentration of LNFPI would show antimicrobial activity. Using area under the curve analyses, we found that over 24 h, LNFPI had no significant effect, although there was an apparent decrease in growth at 20 mg/mL (Figure 5G). LNFPIII, though, significantly reduced the growth of COH1 when dosed at 7.5, 10, 15, and 20 mg/mL (8.78 mM, 11.7 mM, 17.6 mM, and 23.4 mM) (*p* = 0.0259, *p* = 0.0169, *p* = 0.0150, *p* = 0.0003, respectively via one-way ANOVA with Dunnett’s post hoc test; Figure 5G). For both LNFPI and LNFPIII, 7.5

## Discussion

The rise of multidrug resistant bacteria underscores the urgent need for novel antibiotics and new protein targets. Current antibiotics, such as β-lactams and glycopeptides, remain among the most effective and extensively used classes, as they inhibit key stages of cell wall synthesis. More recently, PG hydrolases have also emerged as promising antibacterial targets. Indeed, the Wuest group discovered that a carolacton analog binds to and inhibits GbpB, a homologue of PcsB, in *Streptococcus mutans*.^66^ In addition, direct binding to PG can also inhibit hydrolase function, as demonstrated by complestatin and corbomycin.^67^ These examples highlight that multiple components of the PG machinery, including essential proteins like PcsB, represent viable and attractive targets for antibiotic development.

Herein, we identified PcsB, an essential PG hydrolase in GBS, as a potential interaction partner for HMOs. PcsB was initially explored due to its downregulation in the global untargeted proteomics data set. However, this decrease in PcsB abundance does not align with the observed chain truncation, as septal PG hydrolysis and daughter cell separation typically represent the final steps of the cell division process. Closer inspection of our RNA-seq data revealed differential expression of multiple genes involved in cell division and septal remodeling, including the increased expression of *ftsA* and *pbp2b*, alongside decreased expression of *ftsE*, and changes in Mur pathway genes. Interestingly, a muramoyltetrapeptide carboxypeptidase, SAG1889, displayed decreased protein abundance despite increased transcript levels, supporting the presence of disrupted post-transcriptional regulation with HMO treatment. The combined transcriptional changes observed suggest a coordinated perturbation of septal maturation and cell wall remodeling, rather than a simple defect in PG synthesis or cell division alone.

Taken together, these findings suggest multiple, nonexclusive hypotheses. First, the proteomics measurements may not fully capture functional PcsB abundance due to its tight regulation and dependence on FtsEX-mediated activation. Second, the HMO concentration and/or incubation period could have been insufficient to observe predicted increased levels. Finally, GBS may respond to reduced activity of the FtsEX-PcsB axis by varying the expression of mentioned cell division and cell wall remodeling genes, contributing to altered divisome dynamics that could indirectly influence the chain morphology. Given the essential role of PcsB in viability and its extensive characterization in the literature, we prioritized investigating its relevance to HMO-mediated inhibition of GBS.

After successful purification of the active CHAP domain of PcsB, supplementation of this domain resulted in a notable increase in the chain length of GBS. One possible explanation is that the presence of exogenous, tagged CHAP perturbs normal regulation of PG hydrolysis, resulting in reduced expression or activity of native PcsB. Indeed, reduced expression of *pcsB* in *S. pneumoniae* promoted longer chain length,^40,54^ akin to the morphological changes observed herein. Also indirectly, tagged CHAP could be causing a more global shift or mis-regulation of the timing involving daughter cell separation, leading to chain elongation. We also cannot currently rule out that tagged CHAP itself is directly causing the increased chain length and decrease of cell division; however, we believe this third option is less likely, as PcsB functions downstream of divisome assembly to mediate septal PG cleavage during daughter-cell separation.

Subsequently, we found that the exogenous CHAP domain rescued the growth of GBS in the presence of an inhibitory concentration of HMOs. These data showcase a clear connection between HMO-inhibition and PcsB. These results could be due to several contributing factors. *In silico* and experimental binding assays demonstrated that LNFPI and LNFPIII bind CHAP_PcsB_, with *para*-LNnH and LNnH also emerging as candidate binders. Therefore, sequestration of HMOs by exogenous CHAP could reduce their interaction with native PcsB, allowing residual hydrolase activity to commence. Yet the micrographs still demonstrated increased chain length and cell debris under combined HMO and CHAP treatment, indicating that exogenous CHAP exerts effects beyond ligand sequestration. Despite the increase in extracellular debris observed via SEM with tagged CHAP, cell viability indicated no significant decrease. This suggests that the debris does not originate from cell lysis or leakage. Instead, we hypothesize that this material represents liberated cell wall components, enzymatically cleaved by PcsB (native or the tagged CHAP) during the separation process.

Interestingly, the growth rescue occurred only after ∼18 h of incubation. We hypothesize that this delayed recovery can reflect the time required for sufficient HMO sequestration, competition between endogenous and exogenous CHAP for ligand binding, or the gradual rebalancing of cell wall synthesis and hydrolysis pathways. Together, these findings suggest that HMO-mediated inhibition disrupts the FtsEX-PcsB axis and that exogenous CHAP can restore growth without restoring morphology.

To directly test the hypothesis that HMOs inhibit PcsB catalytic activity, we performed a turbidimetric assay using *S. aureus* PG. The addition of HMOs inhibited the cleavage from tagged CHAP in a dose-dependent manner, with significant inhibition observed at 10 mg/mL HMO, but not with 5 mg/mL HMO. This concentration dependence is consistent with the weak binding affinities observed in the MST experiments and suggests that inhibition requires HMO concentrations in the high micromolar to low millimolar range. This biochemical inhibition provides a mechanistic basis for the growth rescue observed with exogenous CHAP. By directly blocking the enzymatic activity of the CHAP domain, HMOs likely impede cell wall remodeling required for GBS viability. Importantly, the observation that tagged CHAP rescues growth despite this inhibition suggests that sufficient excess CHAP can outcompete HMOs for PG substrate or sequester HMOs away from endogenous PcsB.

Leveraging the MD simulations, we identified four HMOs, LNFPI, LNFPIII, *para*-LNnH, and LNnH, to be predicted binders of the CHAP domain. MST data confirmed the binding of LNFPI and LNFPIII, albeit with relatively weak affinities. As LNFPI had no demonstrated activity at any concentration, we conclude that its affinity for PcsB is too weak to exert effects on its own, yet it is probable that even this minimal binding contributes to the antimicrobial action in conjunction with other binders in the heterogeneous HMO cocktail.

Although the *K*_d_ for LNFPIII was ∼20-fold lower than that of LNFPI, the relatively weak binding affinity, 263 ± 72 μM, is still consistent with the results of the antimicrobial susceptibility results. Specifically, LNFPIII exhibited no measurable MIC up to the highest concentration tested (20 mg/mL, 23.4 mM). Only a ∼17% reduction in growth was observed comparing the average growth over 24 h of COH1 in medium alone to media with 7.5 mg/mL (8.78 mM) of LNFPIII. In a previous study, 5.0 mg/mL (5.86 mM) of LNFPIII caused a moderate ∼26% growth reduction against GB590.^64^ These observations of substantial concentrations needed for minimal growth defects validate the poor functional potency expected from a ligand displaying a weak dissociation constant. We also hypothesize that a similar trend of dissociation constants would be seen with LNnH and *para*-LNnH as only the former was shown to have any activity against GB590 at 5 mg/mL. Because the MIC of each generated HMO cocktail varies with the composition and content of HMOs in breast milk, we can infer that a mixture with a lower MIC has higher concentrations of PcsB binders. The requirement for high HMO concentrations to achieve inhibition in the turbidity assay (>5 mg/mL) aligns well with the weak micromolar binding affinities observed by MST. The quantitative agreement between binding affinity and functional inhibition supports a direct mechanism in which HMOs must occupy the CHAP substrate-binding region at high concentrations to effectively block PG hydrolysis.

Together, these striking discoveries reveal that HMOs prevent GBS growth by directly inhibiting the essential cell wall separation machinery catalyzed by PcsB. Further, it provides novel evidence that an HMO, or any structurally defined complex carbohydrate, can directly engage and inhibit a bacterial PG hydrolase. This work positions HMOs as mechanistic probes and potential leads for next-generation antibiotic development.

## Supplementary Material



## Notes

Additional experimental details, materials, and methods are available for GBS bacterial strains and culture conditions, HMO isolation, proteomics sample preparation and tryptic digest, mass spectrometry analysis for proteomics, scanning electron microscopy (SEM), confocal laser scanning microscopy (CLSM), purification of GST-tagged CHAP domain, bacterial growth and viability assays, turbidimetric peptidoglycan hydrolysis assay, structural prediction and molecular docking, molecular dynamics (MD) simulation, purification of His_6_-tagged CHAP domain by GenScript, microscale thermophoresis (MST), and statistical analysis (PDF)

## △

Department of Food Technology Faculty of Technology Rajarata University of Sri Lanka Mihintale 50300 Sri Lanka

## Notes

This study is dedicated to Prof. Samuel J. Danishefsky on the occasion of his birthday.
