# Supplementary material for: Human Milk Oligosaccharides Inhibit Group B Streptococcal Growth by Binding PcsB, an Essential Cell Wall Separation Protein
Source: JACS Au. 2026 Mar 27;6(4):2355–66. doi: 10.1021/jacsau.5c01741 (PMC13126162; doi:10.1021/jacsau.5c01741)
Supplement: Supplementary file 1 [file au5c01741_si_001.pdf]

Supporting Information

Human Milk Oligosaccharides Inhibit Group B Streptococcal Growth by Binding PcsB, an Essential Cell Wall Separation Protein

Julie A. Talbert[1], Thomas L. Kalmer[1], Lee S. Cantrell[2], Mithila D. Bandara,[3] Alexei V. Demchenko[3], Allison S. Walker[1,4], Jennifer A. Gaddy[5, 6, 7], and Steven D. Townsend[1,*]

[1]Department of Chemistry, Vanderbilt University, Nashville, Tennessee 37240, United States
[2]Department of Biochemistry, Vanderbilt University, Nashville, Tennessee, United States
[3]Department of Chemistry, Saint Louis University, St. Louis, Missouri, 63103, United States
[4]Department of Biological Sciences, Vanderbilt University, Nashville, Tennessee, 37235
[5]Department of Medicine, Vanderbilt University Medical Center, Nashville, Tennessee 37232, United States
[6]Department of Pathology, Microbiology and Immunology, Vanderbilt University Medical Center, Nashville, Tennessee 37232, United States
[7]Department of Veterans Affairs, Tennessee Valley Healthcare Systems, Nashville, Tennessee 37212, United States
[*]Denotes Corresponding Authorship

Keywords: Group B *Streptococcus*, PcsB, cell wall separation, microscale thermophoresis, proteomics

Table of Contents

Materials and Methods S2-S7
Supplemental Tables S7
Supplemental Figures S8-S17
References S18

Materials and Methods

GBS bacterial strains and culture conditions. The bacterial strains used in this study were COH1 and GB00590 (GB590). COH1 is a serotype III, multi-locus sequence type 17 that was isolated from human blood and purchased from ATCC. GB590 is a previously characterized strain recovered from the vaginal/rectal swab of a colonized pregnant woman and was classified as multi-locus sequence type 19 with a serotype III polysaccharide capsule.[1] GBS was grown on blood agar plates and in Todd-Hewitt broth (THB) at 37 °C in ambient air. Following incubation, bacterial density was quantified through absorbance readings at 600 nm (OD_{600}) and bacterial numbers were estimated using the predetermined coefficient of 1 $OD_{600} = 10^9$ colony forming unit (CFU)/mL.

HMO isolation. Human milk was obtained from healthy, lactating patients between 3 days and 3 months postpartum and stored between −80 and –20 °C. Deidentified milk was provided by Dr. Jörn-Hendrik Weitkamp from the Vanderbilt Department of Pediatrics, under a collection protocol approved by the Vanderbilt University institutional review board (IRB #100897), or from Medolac. Milk samples were thawed and then centrifuged for 45 min. Following centrifugation, the resultant top lipid layer was removed. The proteins were then removed by diluting the remaining sample with roughly 1:1 (vol/vol) 200 proof ethanol, chilling the sample briefly, and centrifuging for 45 min, followed by removal of the resulting HMO-containing supernatant. Following concentration of the supernatant in vacuo, the HMO-containing extract was dissolved in 0.2 M phosphate buffer (pH 6.5) and heated to 37 °C. β-Galactosidase from Kluyveromyces lactis was added, and the reaction mixture was stirred until lactose hydrolysis was complete. The reaction mixture was diluted with roughly 1:0.5 (vol/vol) 180 or 200 proof ethanol, chilled briefly, and then centrifuged for 30 min. The supernatant was removed and concentrated in vacuo, and the remaining salts, glucose, and galactose were separated from the oligosaccharides using size exclusion chromatography with P-2 gel (H_2O eluent). The oligosaccharides were then dried by lyophilization. HMO isolates from donors were combined and solubilized in water to reach a final concentration of 102.6 mg/mL.

Proteomics sample preparation and tryptic digest. For each experiment, GB590 cultures were established with 2 mg/mL HMO (below minimum inhibitory concentration (MIC)) prior to overnight incubation in 500 µL of Todd-Hewitt broth (THB). Three biological replicates were used for each group. An OD_{600} normalized set of samples were pelleted at 20,000 x g for 15 min prior to 3x wash with PBS. Washed pellets were homogenized in 5% SDS by vortexing, pulse sonication, and boiling for 10 min at 95 °C. A total of 20 ug protein isolate was isolated, as measured by Pierce BCA assay. Protein isolates were digested according to the Protifi S-Trap Micro protocol. Briefly, dissolved protein isolates were reduced by addition of dithiothreitol to 20 mM with incubation at 56 °C for 1 h. Reduced disulfide bonds were alkylated by addition of iodoacetamide to 40 mM, followed by dark, room temperature incubation for 30 min. Proteins were then acidified by addition of phosphoric acid to 1.2% v/v prior to protein precipitation by addition of 600% v/v S-Trap Buffer: 100 mM triethylammonium bicarbonate (TEAB) in 90% MeOH, 7.5 pH. Precipitated proteins were deposited on the S-Trap micro membrane and S-Trap Buffer removed by vacuum filtration. Proteins were further washed by 3x addition of 160 uL S-Trap buffer. Proteins were digested by addition of 750 ng proteomics-grade trypsin in 25 uL 50 mM TEAB, 7.5 pH. S-Traps were capped and incubated at 47 °C, 1 h. Peptides were eluted by 40 uL serial additions of 50 mM TEAB, 0.2% aqueous formic acid, 50 mM TEAB, and 50% acetonitrile. Peptides were dried by vacuum centrifugation and suspended in 0.2% aqueous formic acid prior to analysis.

Mass spectrometry analysis for proteomics. Peptides were individually analyzed using a Dionex Ultimate 3000 UHPLC coupled to a Q-Exactive Plus tandem mass spectrometer (Thermo Scientific, San Jose, CA) by the Vanderbilt Mass Spectrometry Research Center Proteomics Core. Peptides were separated along a 20 cm length in-house pulled capillary column with 100 μm inner diameter, packed with 3 μm C18 resin. Solvent A was 0.1% aqueous formic acid and solvent B was 0.1% formic acid in acetonitrile. Approximately 250 ng peptides were separated at a flow rate of 350 nL/min on a 73-min gradient from 2 to 40% organic phase, followed by a 17-min washing step and a 35-min blank injection between runs. The mass spectrometer was operated in top-15 data-dependent analysis mode with dynamic exclusion. Precursor spectra were collected from 375 to 1600 m/z at 70,000 resolution (AGC target 3e6, max ion injection time of 60 ms). Product scans were performed at 17,500 resolution (AGC target 1e5, max ion injection time of 100 ms, 30 NCE).

Result RAW files were searched in FragPipe (version 20.0) with search done in MSFragger (version 3.8) against the appropriate UniProt canonical reviewed and unreviewed database (*Streptococcus agalactiae* serotype V, 2,107 protein entries, downloaded 8/8/2023).[2] Carbamidomethylation was set as a static modification. Up to three variable modifications were allowed per peptide, searching for methionine oxidation, n-terminal acetylation, n-terminal pyroglutamate formation from glutamine, and n-terminal water loss from glutamic acid. Identified peptides were validated and filtered at 1% false discovery rate with Philospher (version 5.0.0).[3] Filtered peptides were quantified by IonQuant (version 1.9.8) with match between runs inference enabled.[4]

FragPipe results were processed using custom R Scripts. Briefly, contaminant peptides were filtered, normalization was performed by HarmonizR with ComBat parametric inference.[5, 6] Differential expression was done by Welch's unequal variance parametric t-test. All other R functions were from base R or standard CRAN repositories. Result files are available under PRIDE accession number PXD045633 or under MassIVE accession number MSV000092951.

Scanning electron microscopy (SEM). Bacterial growth was analyzed by SEM as previously described.[7] Briefly, GB590 was cultured in THB with 2 mg/mL HMO supplementation in wells containing 12 mm glass coverslips coated with poly-L-lysine (Corning, Bedford MA) at 37 °C in ambient air for 24 h. GB00590 grown in medium alone served as the control. To investigate morphological changes with addition of the CHAP domain, COH1 was cultured in tagged CHAP (10 μM) ± HMOs (15 mg/mL) and tagged CHAP (10 μM) in 48 well plates containing 8 mm glass coverslips coated with poly-L-lysine (Corning, Bedford MA) at 37 °C in ambient air for 24 h. COH1 grown in medium alone as the control.

Planktonic cells were removed by decanting the culture and washing the wells gently with 1X PBS. Samples were fixed with 2.5% glutaraldehyde and 2.0% paraformaldehyde in 0.05 M sodium cacodylate buffer (pH 7.4, Electron Microscopy Sciences, Hatfield, PA) for 24 h prior to dehydration by sequential washing with increasing concentrations of ethanol. After dehydration, samples were dried at the critical point with a Tousimis critical point dryer machine, mounted onto aluminum stubs, and then coated with ca. 20 nm of gold by plasma sputter coating. Subsequently, sample edges were painted with colloidal silver (Electron Microscopy Sciences) to facilitate the dissipation of charge from the sample surface. Samples were imaged on a Zeiss Crossbeam 550 FIB-SEM at 2 keV using the in chamber secondary electron detector, images were acquired using Zeiss SmartSEM software.

Confocal laser scanning microscopy (CLSM). GB590 was cultured in THB with 7 mg/mL HMO (subinhibitory concentration of the current HMO cocktail) in Eppendorf tubes at 37 °C in ambient air for 12 h. GB590 grown in medium alone served as the control. Aliquots (50 µL) of bacterial cultures stained with DAPI (final concentration: 1 mg/mL) and Fl-Van (a 1:1 mixture of vancomycin and Bodipy FL-conjugated vancomycin, final concentration: 1 mg/mL) for 5 min in dark at room temperature, as previously described.[8] Cells were pelleted by centrifugation at 13,000 x g for 5 min and the supernatant was decanted. Samples were resuspended with 100-200 µL of 2.5% glutaraldehyde and 2.0% paraformaldehyde in 0.05 M sodium cacodylate buffer (pH 7.4, Electron Microscopy Sciences, Hatfield, PA) and 20 uL was spread onto 12 mm glass coverslips coated with poly-L-lysine (Corning, Bedford MA). The next day, coverslips were mounted on glass microscope slides using Cytoseal XYL mounting media (Thermo Scientific, Waltham MA). Samples were imaged with a Zeiss LSM 980 confocal laser scanning microscope with Zen 3.3 blue edition software using an oil-immersion 63x/1.4 Plan-Apochromat objective lens. DAPI was imaged with 405 nm excitation and 411-606 nm emission. Bodipy FL-conjugated vancomycin was imaged with 488 nm excitation and 490-650 nm emission. For all images, the pinhole was set to 1 Airy Unit, Pixel Dwell Time was 4.1 µm, Frame size was 2048x2048, and zoom=1. Scaling was 0.066 µm per pixel.

Purification of GST-tagged CHAP domain:

Plasmid transformation and expression in E. coli BL21 Star (DE3). The CHAP domain of the *Streptococcus agalactiae* protein PcsB was cloned into the pGS21a vector by GenScript (Piscataway, NJ). The resulting plasmids encoded an N-terminal GST tag for affinity purification and increased solubility. Plasmids were transformed into chemically competent E. coli BL21 (DE3) cells via heat shock. Briefly, 100 ng of plasmid DNA was added to 50 µL of competent cells, incubated on ice for 30 min, heat-shocked at 42 °C for 10 s, and then returned to ice for 5 min. SOC medium (1 mL) was added to the cells and growth was allowed for 1 h at 37 °C with shaking (220 rpm) before plating on Luria Bertani (LB) agar containing 0 µg/mL, 10 µg/mL, 50 µg/mL, and 100 µg/mL carbenicillin. Plates were incubated overnight at 37 °C. After successful transformation, appropriate colonies were screened by plasmid miniprep (QIAprep Spin Miniprep Kit) and sequence-verified by Sanger sequencing (GenHunter, Nashville, TN) using T7 vector primers. A single verified colony was used to inoculate LB Lennox medium (10 mL) containing 100 µg/mL carbenicillin. Cultures were incubated overnight at 37 °C with shaking (220 rpm). The next day, the 10 mL culture was used to inoculate 2 L of LB Lennox medium containing 100 µg/mL carbenicillin. The culture was incubated at 37 °C with shaking (220 rpm) until it reached an OD_{600} of 0.4–0.6. Protein expression was induced with 0.3 mM IPTG, and the temperature was reduced to 18 °C. Cultures were allowed to express protein overnight under these conditions.

Cell harvest and lysis. The following day, cells were harvested by centrifugation at 4,200 rpm for 1 h at 4 °C. Cell pellets were resuspended in PBS (pH 7.3) at ~10% of the original culture volume. Cells were lysed on ice using a probe sonicator at 100% amplitude with a 5-second on, 5-second off pulse cycle for 30 min. The lysate was clarified by ultracentrifugation at maximum speed (~35,000 × g) for 90 min at 4 °C.

Purification and analysis. The clarified lysate was loaded onto a GSTrap FF affinity column (Cytiva) pre-equilibrated in PBS (pH 7.3) using an ÄKTA FPLC system. The column was washed PBS (pH 7.3) and bound GST-tagged CHAP domain was eluted using 50 mM Tris-HCl, 10 mM L-glutathione reduced, pH 8 elution buffer using a flow rate of 2.0 mL/min. Elution was monitored by UV absorbance at 280 nm, and peak-containing fractions were pooled accordingly. The pooled sample was concentrated using 3 kDa molecular weight cut-off, polyethersulfone (PES) protein concentrators (ThermoScientific Pierce) and the buffer was switched to 10% glycerol (v/v) in PBS. Pooled fractions were filtered through 0.22 µM PES syringe filters and analyzed by SDS-PAGE to assess purity. Western blot was performed using an anti-GST mAB (Invitrogen, CatNo: MA4-004-HRP).

Bacterial growth and viability assays. COH1 was grown overnight as described above and used to inoculate fresh THB at a multiplicity of infection (MOI) of $5X10^5$ colony forming units per 40 µL of growth medium in 384 well tissue culture treated, sterile polystyrene plates (Greiner Bio-One). HMOs were dissolved in DI water to achieve a concentration of 102.6 mg/mL and filtered through a 0.22 µm syringe filter. All proteins (tagged CHAP and GST) were kept in 10% glycerol (v/v) in PBS at ~40 µM stocks. Proteins were added to achieve a final concentration of ca. 10 µM protein per well. HMOs were added to achieve a final carbohydrate concentration of ca. 15 mg/mL, the MIC of the current HMO cocktail. Bacteria grown in THB in the absence of any HMOs or protein served as the control. Cultures were grown at 37 °C in ambient air for 24 h. Growth was quantified through spectrophotometric reading at 600 nm. At 24 h, viability was assessed via serial dilutions of culture, plating into blood agar plates, and quantifying colonies.

Turbidimetric peptidoglycan hydrolysis assay. The enzymatic activity of the CHAP domain of PcsB was evaluated via a light-scattering assay monitoring the hydrolysis of *S. aureus* peptidoglycan (PG) (Sigma-Aldrich). PG was suspended in assay buffer (50 mM Tris-HCl, 100 mM NaCl, pH 8.0) to a final concentration of 2 mg/mL. To ensure a homogenous substrate environment, the suspension was subjected to 60 seconds of high-speed vortexing. Prior to the assay, the purified CHAP domain (40 µM stock) and a vehicle control (PBS, 10% glycerol) were reduced with 5 mM dithiothreitol (DTT) for 20 minutes on ice to ensure the catalytic availability of the active-site cysteine. Assays were performed in a 384-well plate. Master mixes were prepared to achieve a final CHAP concentration of 15 µM, with HMOs at 5 mg/mL and 10 mg/mL. The reaction was initiated by adding the PG substrate last. Turbidity was monitored by measuring the OD_{600} at room temperature using a microplate reader. To maintain the insoluble PG in suspension, continuous orbital shaking was performed prior to each kinetic read. Measurements were recorded every 5 min for a total duration of 3 h. To standardize lytic curves, data are reported as the percent change in OD_{600}.

Structural prediction and molecular docking. The structure for the CHAP domain of PcsB from GBS strain 2603 V/R was predicted using AlphaFold2_advanced Python notebook that was run on Google Colaboratory cloud computing facilites.[9, 10] Structures for all single-entity HMOs and the peptide stem fragment were made in ChemDraw (version 22.2.0) and converted to 3D structures and optimized using the MMFF94 force field in Avogadro (version 1.2.0).[11] Docking was performed using AutoDock Vina (version 1.1.2) with a grid box large enough to cover the entire protein domain.[12, 13] All visualization was performed using PyMOL (version 2.5.7).

Molecular dynamics (MD) simulation. MD simulations were carried out using AmberTools22 and the Amber22 suite. Starting structures for the simulations were HMOs docked to the predicted CHAP domain structure from PcsB (see "Structural prediction and molecular docking for details"). Ligand geometry was extracted from the previously docked structures, and after determining net HMO charge using GaussView 5.0, ligands were parameterized in Antechamber using the options -c bcc -nc (charge from GaussView 5.0) -at gaff2. H++ was used to assign correct protonation states to amino acids in the CHAP domain at a pH of 6.5. Using Xleap, force fields ff19SB and GAFF2 were applied to the protein and ligand, respectively. Additionally, the system was solvated with an SPC/E water model in a truncated octahedral box with a buffer of 14 Å and neutralized using sodium and chlorine ions. After extracting the coordinate and parameter files from Xleap, minimization, equilibration, and heating phases were performed on the system. First, a steepest descent solvent minimization was performed for 500 steps with a restraining force of 500.0 kcal/mol $Å^2$ on the protein-ligand complex, followed by 500 steps of a conjugate gradient minimization.

This process was repeated for the entire system with no restraining force applied to the protein-ligand complex with 1000 steps of steepest descent minimization followed by 1500 steps of conjugate gradient descent. Following the minimization phase was an equilibration phase to heat the system. Heating of the system from 0 to 300 K was done using the Langevin temperature scheme and a 10.0 kcal/mol Å–2 constraint on the protein and ligands over a 20 ps simulation. Finally, equilibration was completed with a constant pressure and temperature of 1 atm and 300 K for 100 ps. Ten replicates of explicit solvent MD simulations were continued at constant pressure for each protein-ligand complex for 19 ns. The time step was set to 2 fs with the trajectory snapshots saved at every 5 ps.

Purification of His_6-tagged CHAP domain by GenScript:

Plasmid transformation and expression in E. coli BL21 Star (DE3). The DNA sequence of the CHAP domain was sent to GenScript (Piscataway, NJ) for optimization, synthesis, and cloning into a pET-22b (+) containing a His6 tag with an added *pelB* sequence. *E. coli* strain BL21 Star (DE3) was transformed with recombinant plasmid. A single colony was inoculated into LB medium containing related antibiotic; culture was incubated in 37 °C at 200 rpm and then induced with 0.5 mM IPTG. SDS-PAGE was used to monitor the expression. Recombinant BL21 Star (DE3) stored in glycerol was inoculated into TB medium containing related antibiotic and cultured at 37 °C. When the OD600 reached about 1.2, cell culture was induced with 0.5 mM IPTG at 37 °C for 4 h.

Cell harvest and lysis. Cells were harvested by centrifugation and pellets were resuspended with lysis buffer (50 mM Tris-HCl, 150 mM NaCl, pH 8.0) followed by sonication. The pellet of cell lysate was solubilized in 50 mM Tris-HCl, 7 M Guanidine hydrochloride, pH 8.0.

Purification and analysis. Target protein was obtained by one-step purification using Ni column. The solubilized sample was loaded onto gravity Ni column and washed with buffer (50 mM Tris-HCl, 8 M Urea, pH 8.0). Then, the His-tagged protein was eluted with buffer (50 mM Tris-HCl, 8 M Urea, pH 8.0 containing imidazole at the final concentration of 20 mM, 50 mM, 500 mM). Then the 500 mM imidazole-eluted protein was refolded by dialysis into final buffer: 50 mM Sodium acetate, 10% glycerol, pH 5.0. The final protein was sterilized with 0.22 μm filter before storage in aliquots. The concentration was determined by Micro-Bradford assay with BSA as standard. The purity and molecular weight were determined by SDS-PAGE and Western blot.

Microscale thermophoresis (MST).

Protein labeling and ligand preparation. The His6-tagged CHAP domain (purified by GenScript as described above) was labeled using the "Monolith His-Tag Labeling Kit RED-tris-NTA 2nd Generation" kit and associated manual (NanoTemper Technologies) at a concentration of 400 nM in PBS with 0.05% Tween 20 (PBS-T) supplied by the kit. LNFPI was prepared as a 240 mM stock solution in PBS-T and 16, 1:2 serial dilutions in PBS-T were made giving concentrations ranging from 120 mM to 7.32 μM. LNFPIII was prepared as a 120 mM stock solution in PBS-T and 16, 1:2 serial dilutions in PBS-T were made giving concentrations ranging from 60 mM to 1.83 μM. Each serial dilution of ligand was mixed with one volume of labeled protein, resulting in a final concentration of 200 nM of labeled CHAP domain and ligand concentrations ranging from 120 mM to 3.66 μM for LNFPI and 60 mM to 1.83 μM for LNFPIII.

Equilibrium timepoint determination. To determine the time at which equilibrium is reached, we used four timepoints at 15 minutes, 60 minutes, 105 minutes, and 150 minutes. At each timepoint, an aliquot from each serial dilution was removed and centrifuged at 10,000 x g for 10 min at 4 °C. Each sample was loaded into Monolith NT.115 [Premium] Capillaries (NanoTemper Technologies) and MST measurements were

performed using a Monolith NT.115 at ambient temperature using the auto-detected power. At each timepoint, the bound value, measured by the MO.affinity analysis software version 2.3 (NanoTemper Technologies), was recorded. The bound values at each timepoint were divided by the bound value measured at 150 minutes to give the fraction bound value.

Dissociation constants determination. Using an incubation of 10-15 minutes, the K_d values for three independently pipetted runs were analyzed and calculated by MO.affinity analysis software version 2.3 (NanoTemper Technologies) for LNFPI and LNFPIII. The K_d values and standard deviations from the three independently pipetted runs for each HMO were subjected to inverse-variance weighted mean to yield the reported K_d ± SD. Fraction bound values computed as negative were normalized to zero after the K_d was calculated.

Statistical analysis. All data shown signify three independent biological experiments each with two or three technical replicates. Data are expressed as the mean ± SEM. Statistical analyses were performed in GraphPad Prism Software v. 10.0.3 (GraphPad Prism Software Inc., La Jolla, California).

Supplemental Tables

Table S1. A recount of the metabolic pathway enrichment analysis.

Pathway	Total Metabolites	Hits	Raw p
Linoleic Acid Metabolism	15	6	2.42E-05
Sphingolipid Metabolism	25	6	0.000595
Glycerophospholipid Metabolism	39	7	0.001334
Pyrimidine Metabolism	60	8	0.004362

Table S2. Kinetic MST results.

HMO (replicates)	Incubation Time (min)	Bound Values[a]	Fraction Bound[a]	K_d [b]
LNFPI (3)	15	865.281	0.990	5.29 ± 1.23
	60	870.618	0.996	5.27 ± 1.58
	105	872.032	0.997	4.45 ± 2.19
	150	873.904	1	6.68 ± 4.45
LNFPIII (1)	15	854.896	0.990	-
	60	864.295	1.001	-
	105	860.092	0.996	-
	150	862.878	1	-

[a]averaged values for LNFPI

[b] K_d not calculated due to single replicate

Supplemental Figures

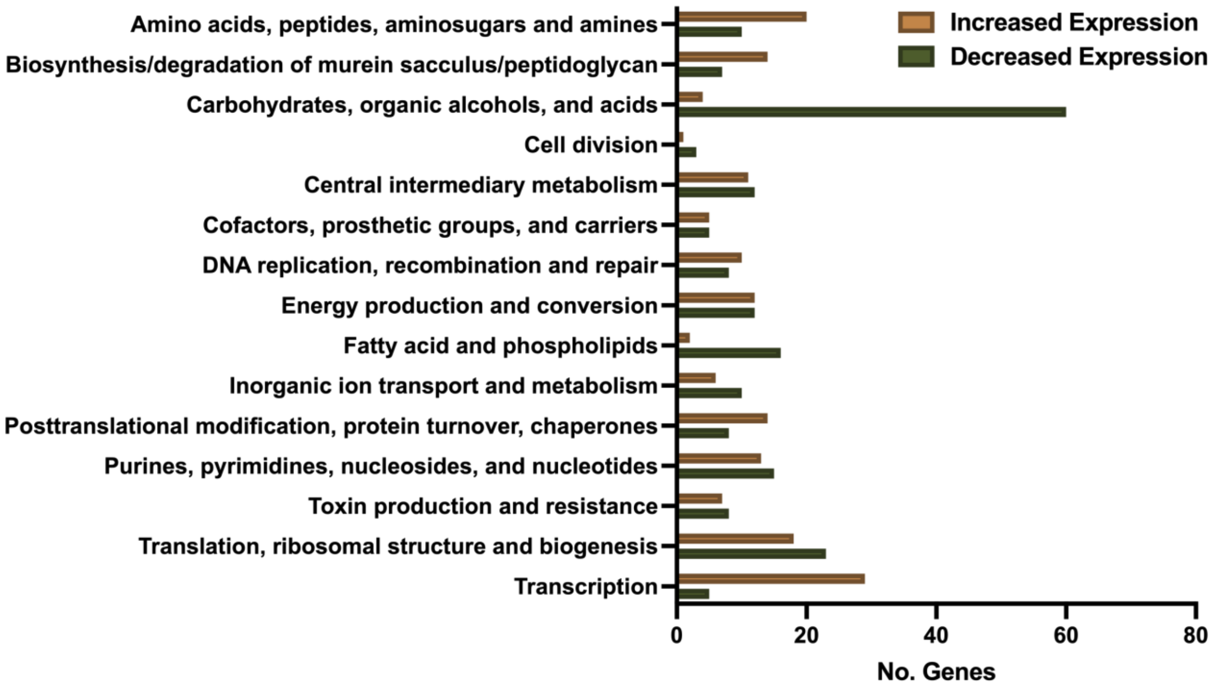

Figure S1. A previous study[7] revealed significant changes in transcript abundance in GBS with exposure to HMOs.

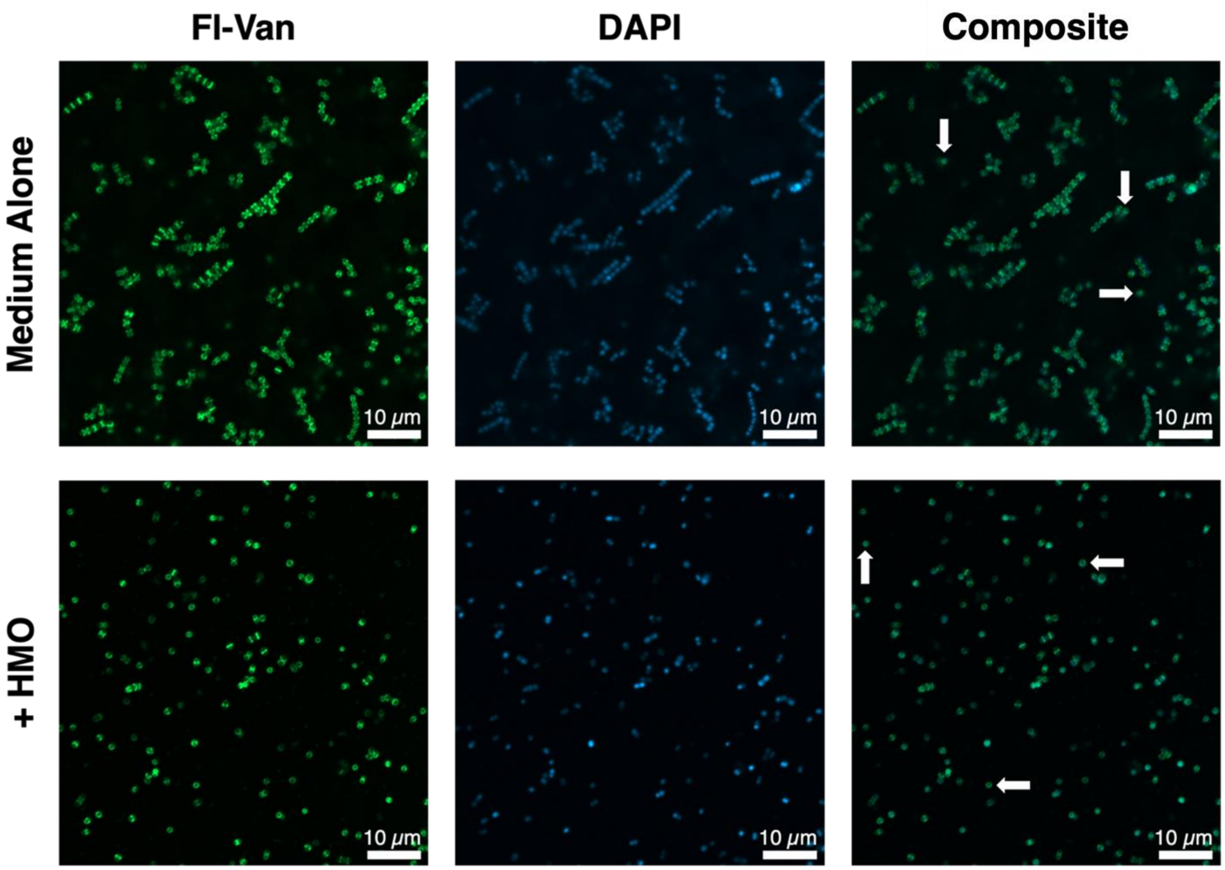

Figure S2. Confocal laser scanning microscopy indicates truncation of chain length and possible cell wall separation perturbation of GBS cells upon HMO exposure. Singular GBS cells, in both untreated and treated samples, show diffuse fluorescent vancomycin (Fl-Van) staining. GB590 cells were also stained with DAPI to view overall cell structure.

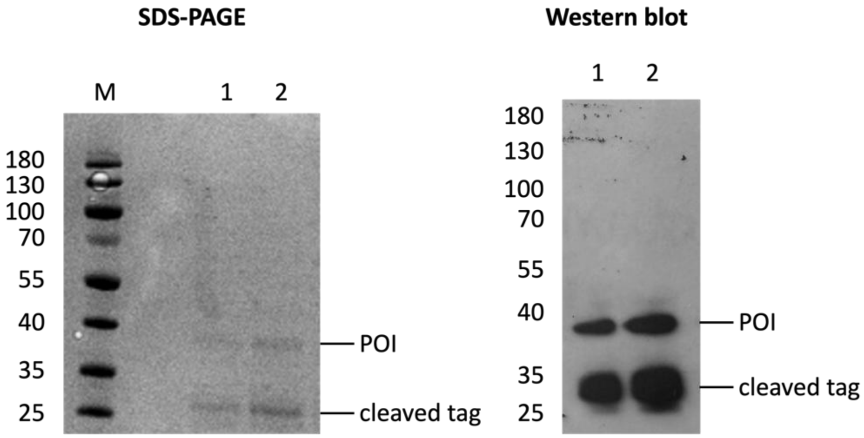

Figure S3. Protein purification of tagged CHAP domain. Lane M: protein marker, Lane 1: 0.5 μg of tagged-CHAP, Lane 2: 1 μg of tagged-CHAP. Western blot was performed with primary antibody: mouse-anti-GST mAB (Invitrogen, CatNo: MA4-004-HRP).

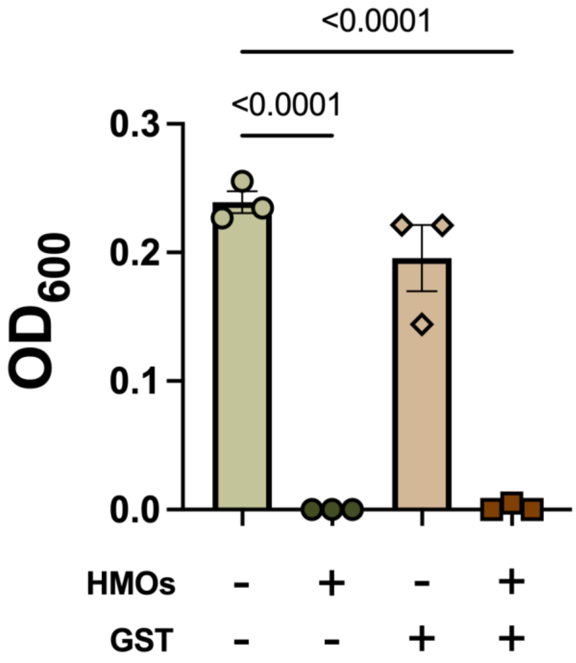

Figure S4. GST is unable to rescue growth of GBS in presence of HMOs. Growth analysis at 24 h demonstrates that HMOs completely inhibit the growth of GBS alone and in combination with GST (P values on graph calculated via one-way ANOVA with Dunnett's post hoc test, N=3). Symbols indicate mean ± SEM.

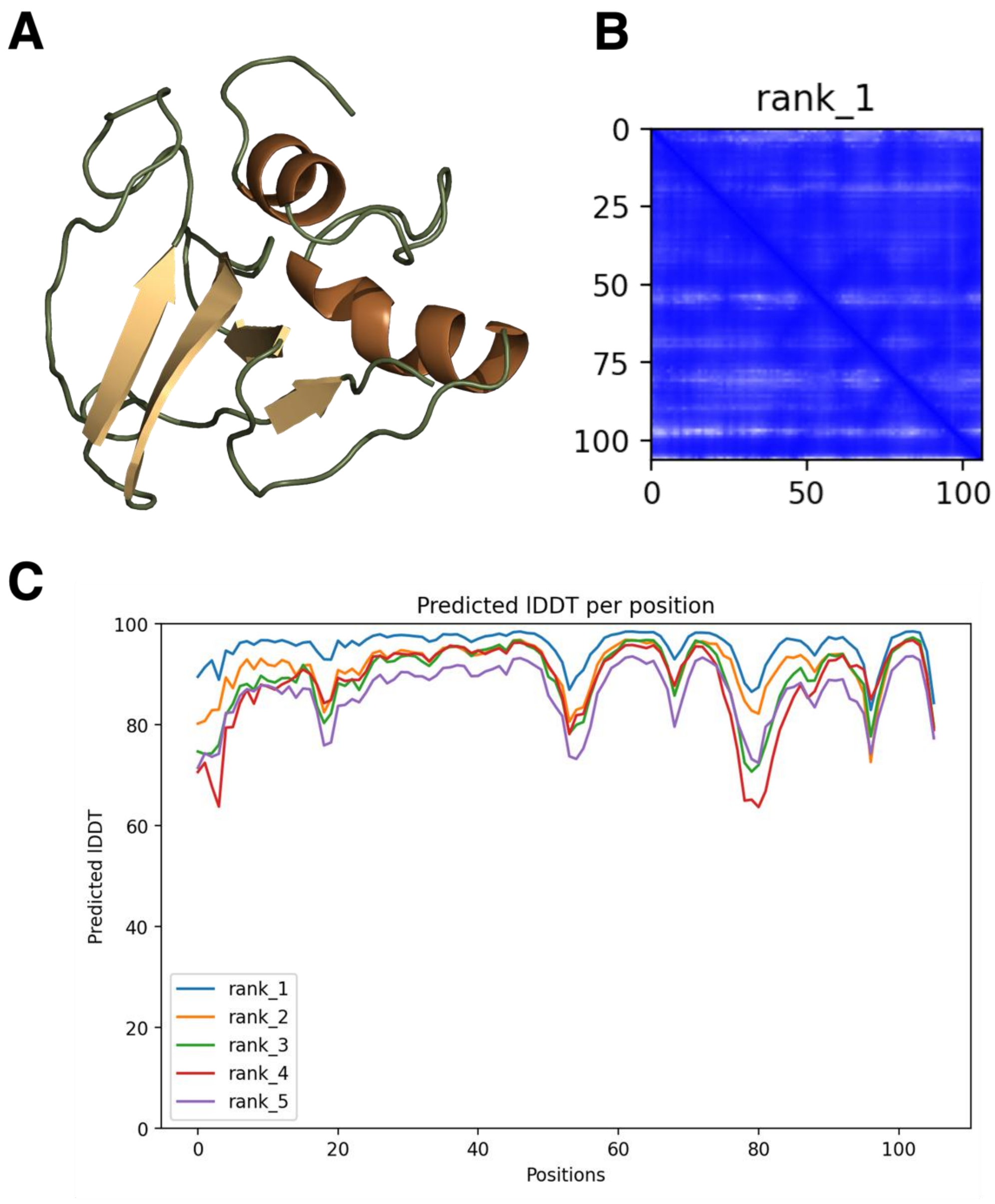

D

Score	Number
pLDDT	95.52
pTM	0.8813

Figure S5. Confidence prediction from AlphaFold. (A) Cartoon visualization of $CHAP_{PcsB}$ predicted by AlphaFold with loops shown in green, helices in brown, and β-sheets in yellow. (B) Predicted alignment error figure detailing high confidence for the CHAP domain. (C) Predicted lDDT per residue position. The model used in our studies was rank 1 (blue) illustrating high lDDT across all residues. (D) High pLDDT and pTM scores predicted via AlphaFold showing confidence in simulated protein.

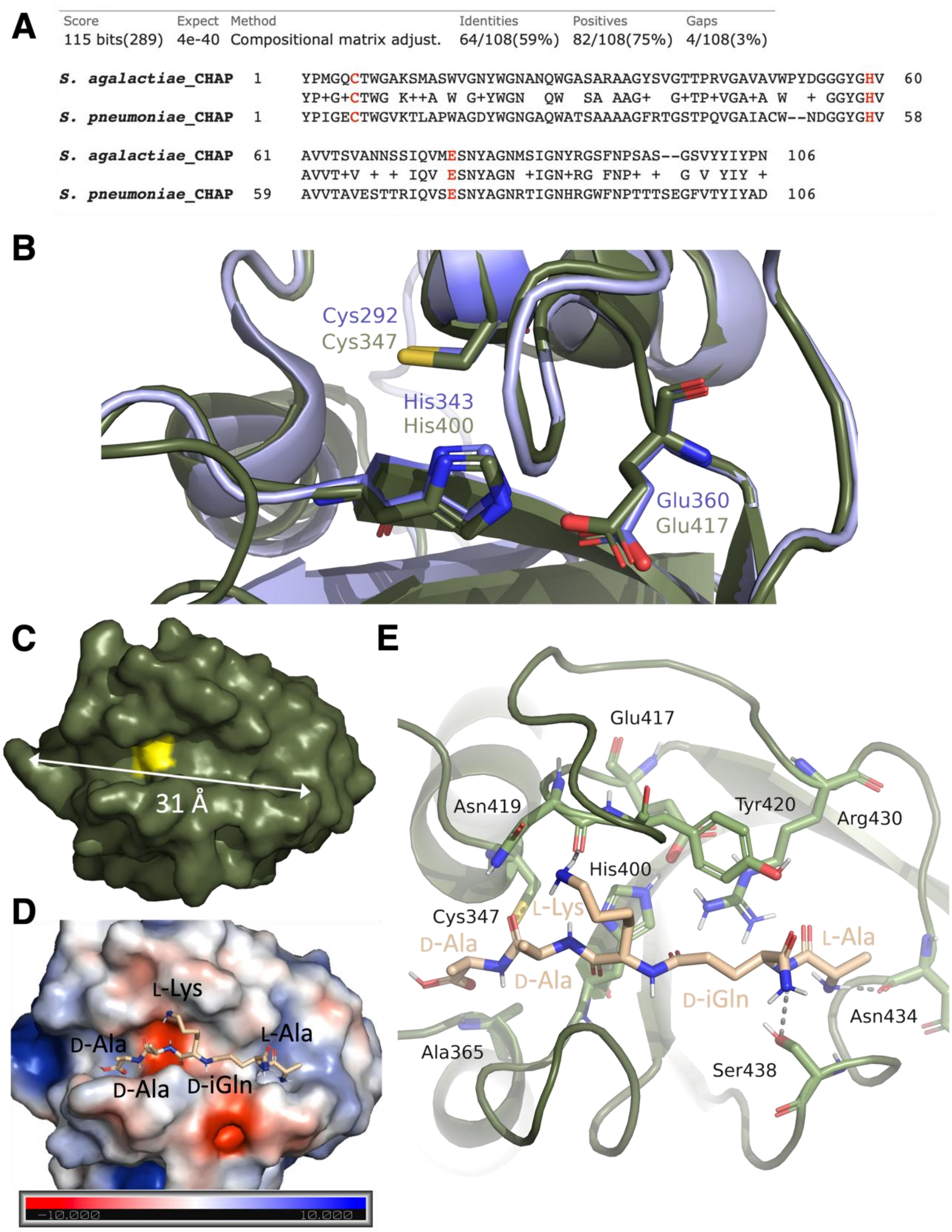

Figure S6. CHAP domain alignment and predicted docking of native PG peptide. (A) Sequence alignment illustrates the catalytic triad of $CHAP_{PcsB}$ of *S. pneumoniae* aligns with the same residues in that of GBS. Catalytic triad residues depicted in red. (B) Visual alignment shows overlap in catalytic triad of $CHAP_{PcsB}$ of *S. pneumoniae* (purple) with that of GBS (green).[14] (C) Molecular surface representation of $CHAP_{PcsB}$. (D) Electrostatic potential surface representation of $CHAP_{PcsB}$ with the pentapeptide, L-Ala-D-iGln-L-Lys-D-Ala-D-Ala, docked. (E) Pentapeptide stabilization interactions in the PcsB active site. Relevant residues are colored green and labeled. Hydrogen bond interactions are depicted as dark gray dotted lines.

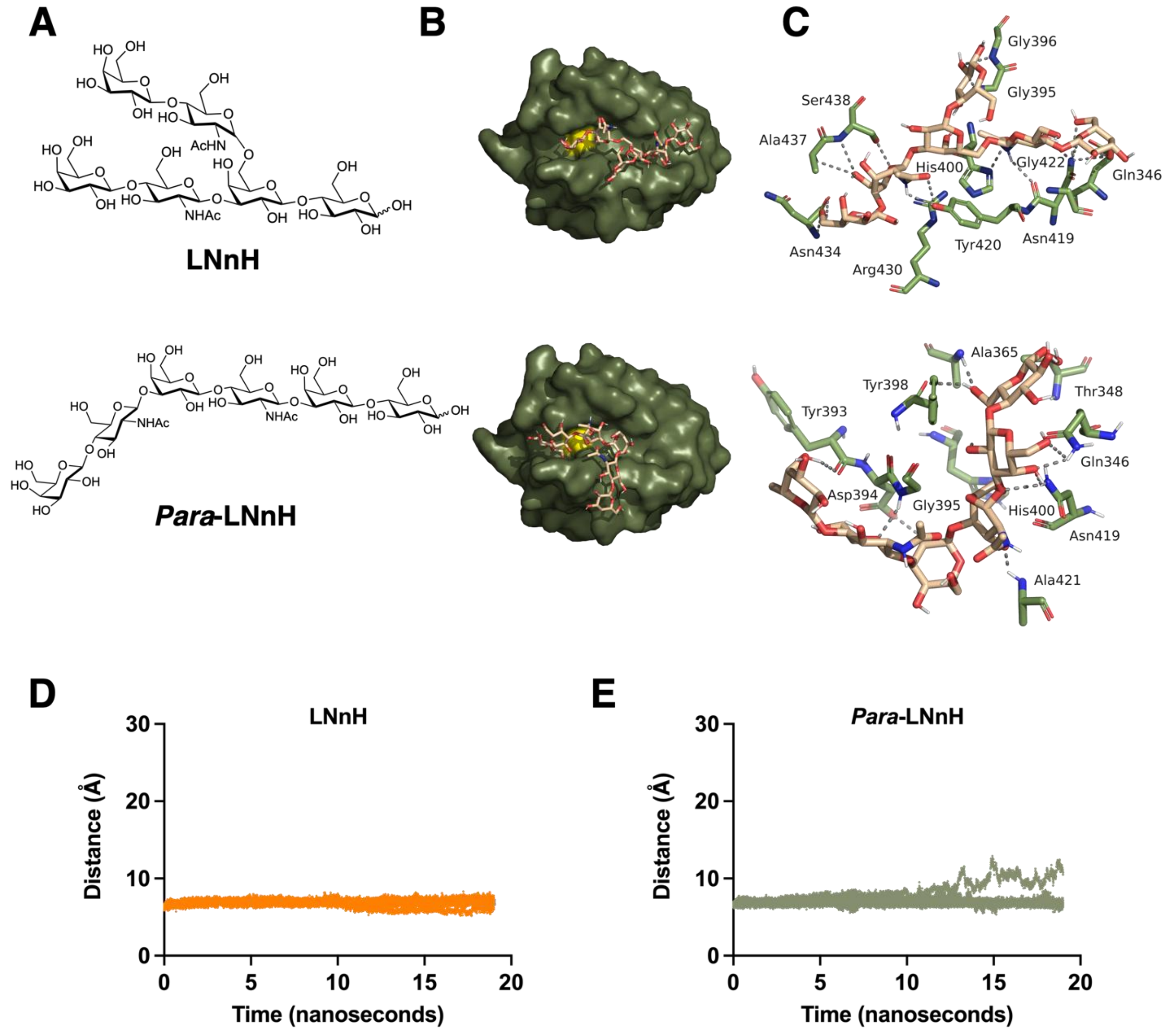

Figure S7. Lacto-*N*-neohexaose (LNnH) and *para*-LNnH are two other predicted binders of $CHAP_{PcsB}$. (A) Structures of LNnH and *para*-LNnH. (B) Docking reveals that both LNnH and *para*-LNnH are predicted to dock in the active site of PcsB. (C) Interactions of LNnH and *para*-LNnH with residues in active site. MD simulation data (10 replicates) demonstrating that LNnH (D) and *para*-LNnH (E) are predicted to remain in the active site of PcsB over 19 ns.

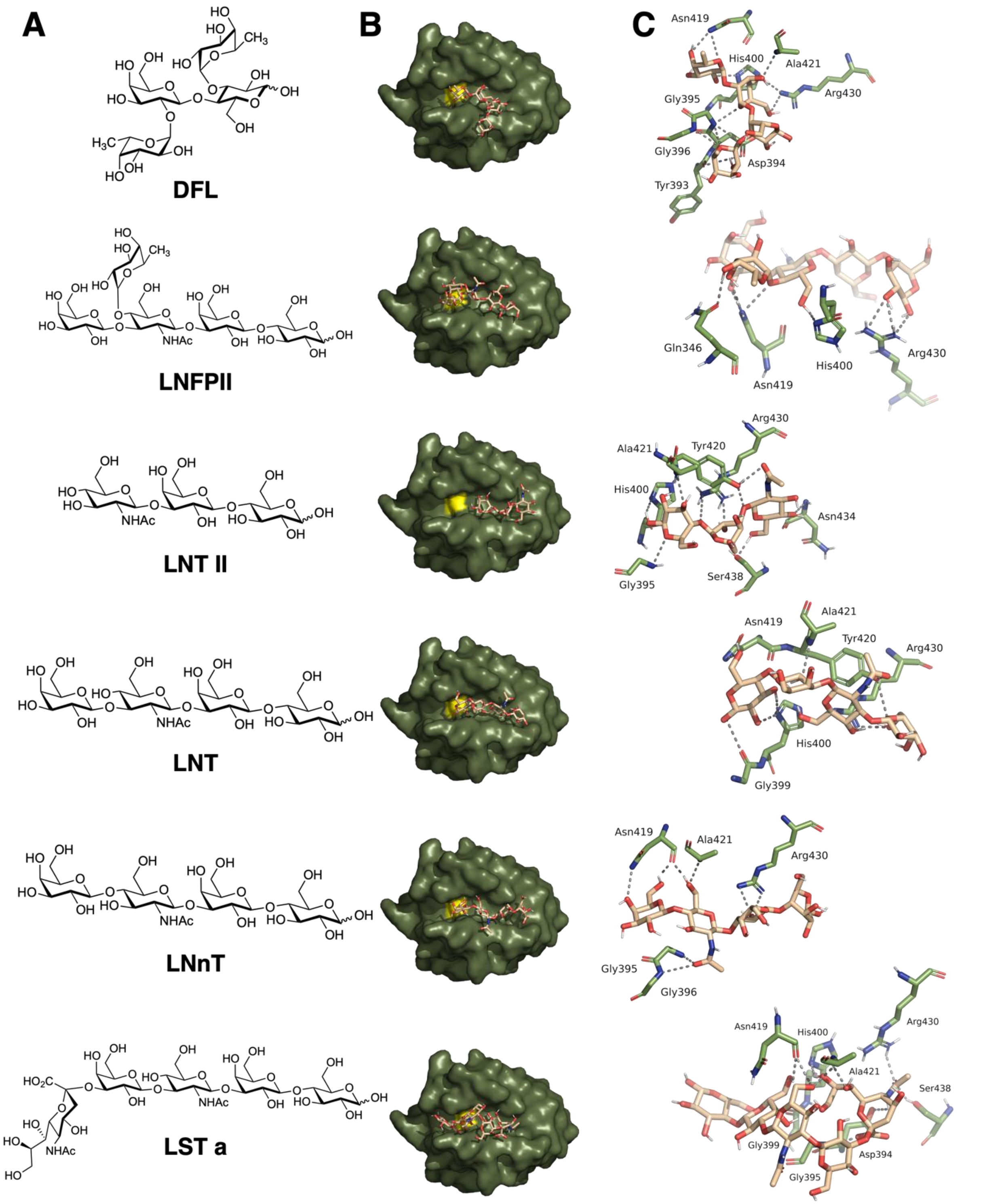

Figure S8. Molecular docking results showing six active HMOs, difucosyllactose (DFL), lacto-*N*-fucopentaose II (LNFPII), lacto-*N*-triose II (LNTII), lacto-*N*-tetraose (LNT), lacto-*N*-neotetraose (LNnT), and LS-tetrasaccharide a (LST a). (A) Structures of DFL, LNFPII, LNTII, LNT, LNnT, and LST a. (B) Docking reveals all active HMOs dock in the active site of PcsB. (C) Interactions of DFL, LNFPII, LNTII, LNT, LNnT, and LST a with neighboring residues.

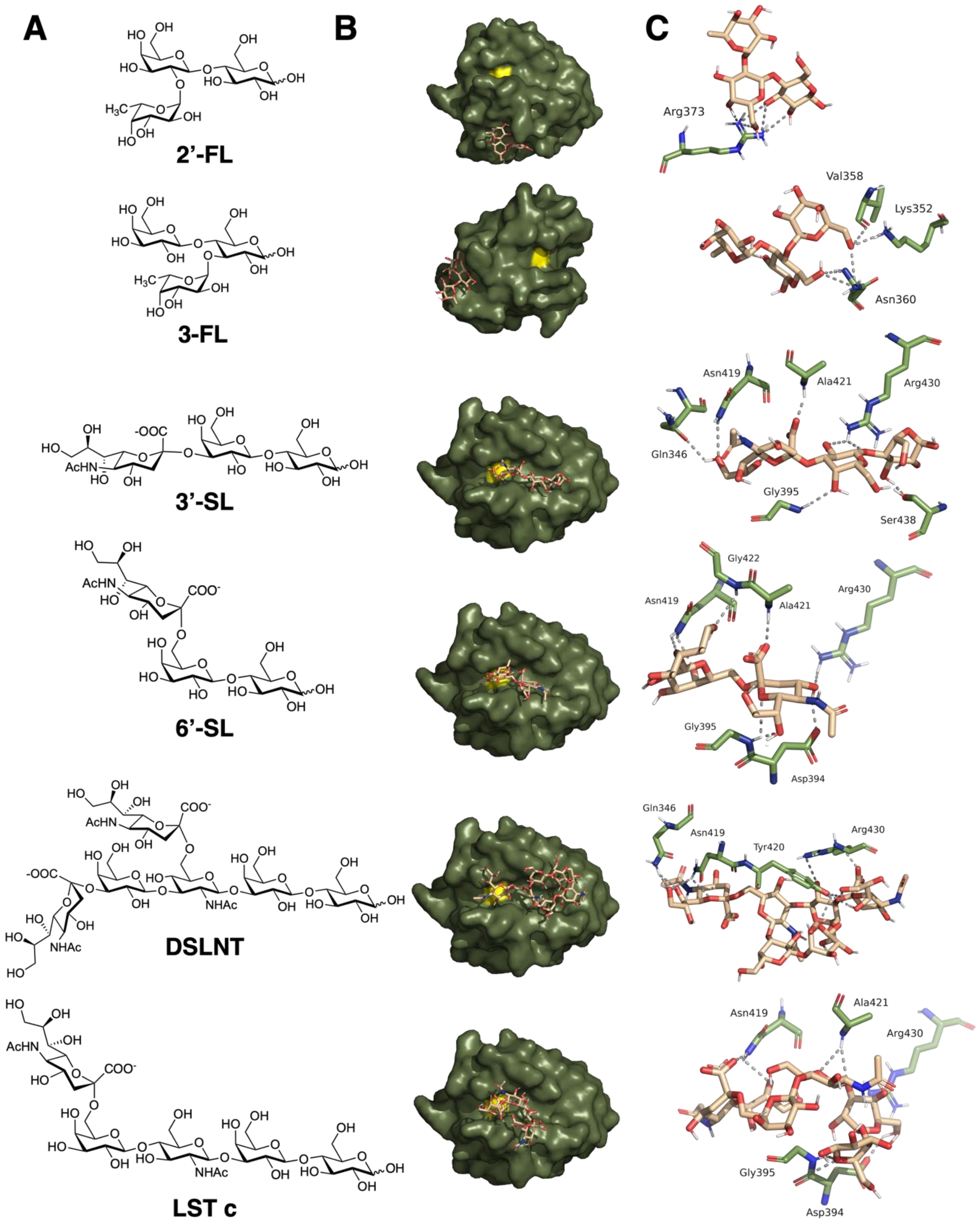

Figure S9. Molecular docking results showing six inactive HMOs, 2'-fucosyllactose (2'-FL), 3-fucosyllactose (3-FL), 3'-sialyllactose (3'-SL), 6'-sialyllactose (6'-SL), disialyllactose-*N*-tetraose (DSLNT), and LS-tetrasaccharide c (LST c). (A) Structures of 2'-FL, 3-FL, 3'-SL, 6'-SL, DSLNT, and LST c. (B) Docking reveals 2'-FL and 3-FL do not dock in the active site of PcsB while 3'-SL, 6'-SL, DSLNT, and LST c do. (C) Interactions of 2'-FL, 3-FL, 3'-SL, 6'-SL, DSLNT, and LST c with neighboring residues.

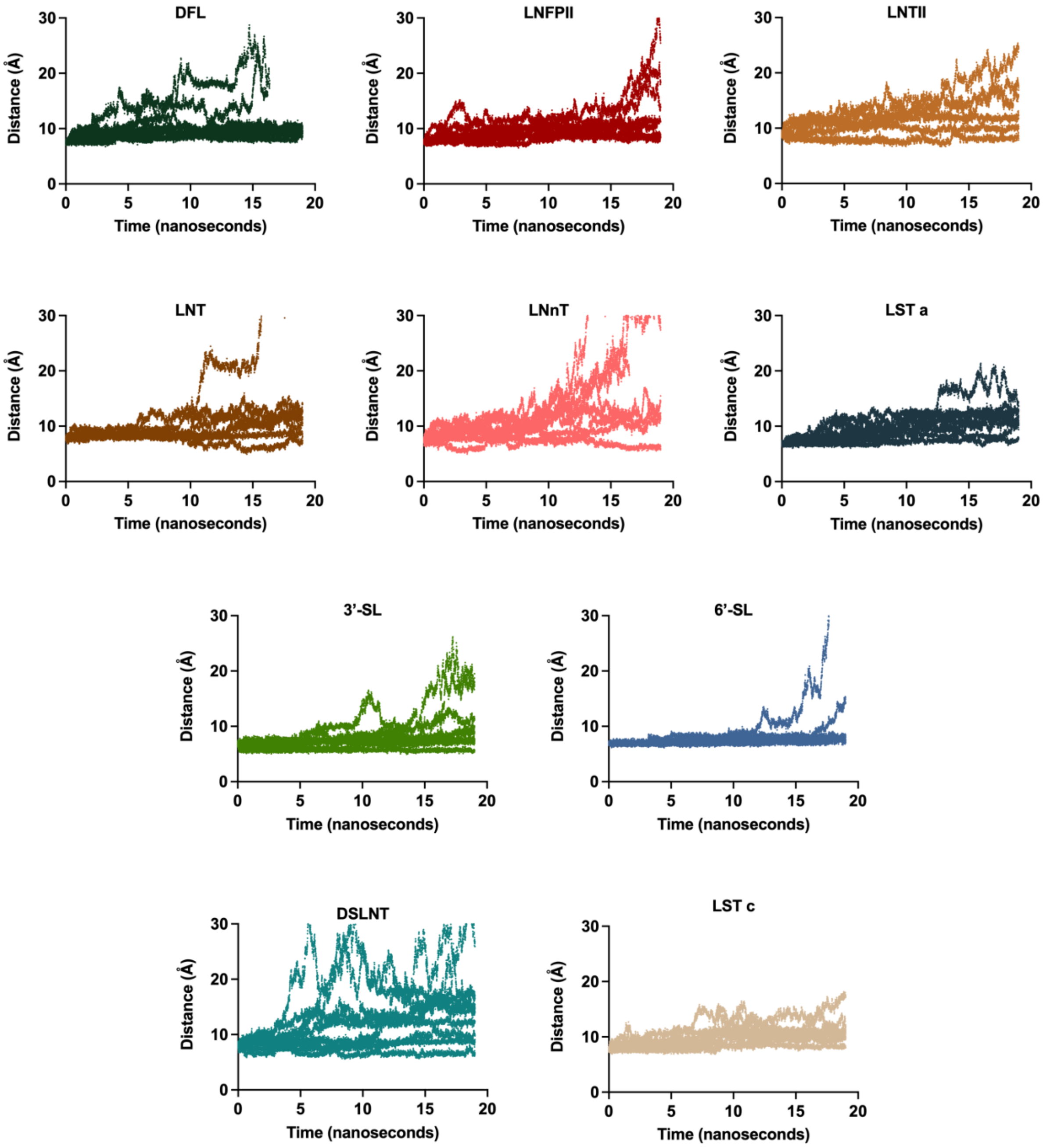

Figure S10. Molecular dynamic simulation data (10 replicates) for DFL, LNFPII, LNTII, LNT, LNnT, LST a, 3'-SL, 6'-SL, DSLNT, and LST c. The top six HMOs have activity against GBS but are not predicted to exert this activity via PcsB binding.

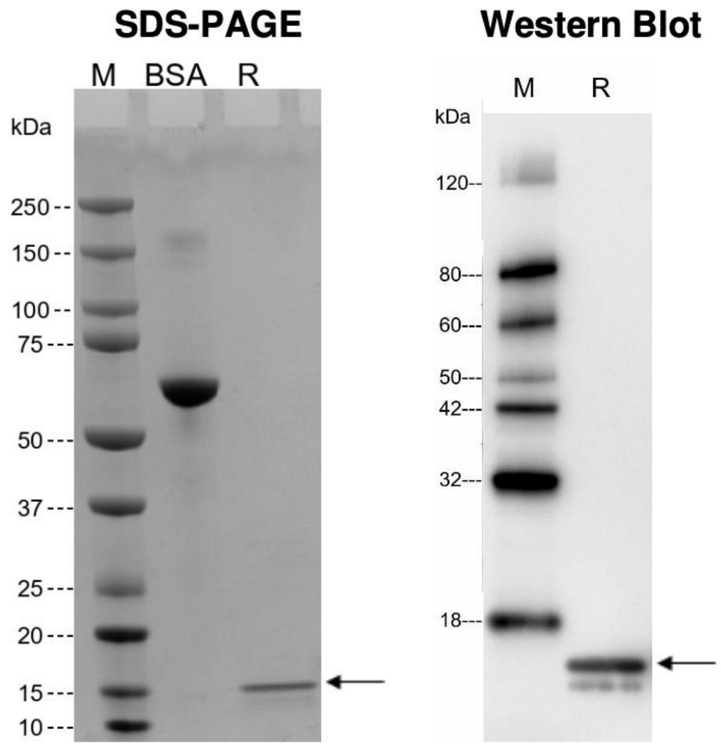

Figure S11. Protein purification via GenScript for the His_6-tagged CHAP domain. Lane M: protein marker, Lane BSA: 2.00 μg as a control, R: reducing conditions for purified protein. Western blot was performed with primary antibody: mouse-anti-His mAb (GenScript, CatNo: A00186).

References:

(1) Manning, S. D.; Lewis, M. A.; Springman, A. C.; Lehotzky, E.; Whittam, T. S.; Davies, H. D. Genotypic diversity and serotype distribution of group B *Streptococcus* isolated from women before and after delivery. *Clin Infect Dis* **2008**, *46* (12), 1829-1837.
(2) Kong, A. T.; Leprevost, F. V.; Avtonomov, D. M.; Mellacheruvu, D.; Nesvizhskii, A. I. MSFragger: ultrafast and comprehensive peptide identification in mass spectrometry–based proteomics. *Nature Methods* **2017**, *14* (5), 513-520.
(3) da Veiga Leprevost, F.; Haynes, S. E.; Avtonomov, D. M.; Chang, H.-Y.; Shanmugam, A. K.; Mellacheruvu, D.; Kong, A. T.; Nesvizhskii, A. I. Philosopher: a versatile toolkit for shotgun proteomics data analysis. *Nature Methods* **2020**, *17* (9), 869-870.
(4) Yu, F.; Haynes, S. E.; Nesvizhskii, A. I. IonQuant Enables Accurate and Sensitive Label-Free Quantification With FDR-Controlled Match-Between-Runs. *Mol Cell Proteomics* **2021**, *20*, 100077.
(5) Voß, H.; Schlumbohm, S.; Barwikowski, P.; Wurlitzer, M.; Dottermusch, M.; Neumann, P.; Schlüter, H.; Neumann, J. E.; Krisp, C. HarmonizR enables data harmonization across independent proteomic datasets with appropriate handling of missing values. *Nat Commun* **2022**, *13*.
(6) Johnson, W. E.; Li, C.; Rabinovic, A. Adjusting batch effects in microarray expression data using empirical Bayes methods. *Biostatistics* **2007**, *8* (1), 118-127.
(7) Moore, R. E.; Spicer, S. K.; Lu, J.; Chambers, S. A.; Noble, K. N.; Lochner, J.; Christofferson, R. C.; Vasco, K. A.; Manning, S. D.; Townsend, S. D.; Gaddy, J. A. The Utility of Human Milk Oligosaccharides against Group B *Streptococcus* Infections of Reproductive Tissues and Cognate Adverse Pregnancy Outcomes. *ACS Cent Sci* **2023**, *9* (9), 1737-1749.
(8) Ng, W. L.; Kazmierczak, K. M.; Winkler, M. E. Defective cell wall synthesis in *Streptococcus pneumoniae* R6 depleted for the essential PcsB putative murein hydrolase or the VicR (YycF) response regulator. *Mol Microbiol* **2004**, *53* (4), 1161-1175.
(9) Jumper, J.; Evans, R.; Pritzel, A.; Green, T.; Figurnov, M.; Ronneberger, O.; Tunyasuvunakool, K.; Bates, R.; Žídek, A.; Potapenko, A.; et al. Highly accurate protein structure prediction with AlphaFold. *Nature* **2021**, *596* (7873), 583-589.
(10) Mirdita, M.; Schütze, K.; Moriwaki, Y.; Heo, L.; Ovchinnikov, S.; Steinegger, M. ColabFold: making protein folding accessible to all. *Nature Methods* **2022**, *19* (6), 679-682.
(11) Hanwell, M. D.; Curtis, D. E.; Lonie, D. C.; Vandermeersch, T.; Zurek, E.; Hutchison, G. R. Avogadro: an advanced semantic chemical editor, visualization, and analysis platform. *J Cheminf* **2012**, *4*.
(12) Eberhardt, J.; Santos-Martins, D.; Tillack, A. F.; Forli, S. AutoDock Vina 1.2.0: New Docking Methods, Expanded Force Field, and Python Bindings. *JCIM* **2021**, *61* (8), 3891-3898.
(13) Trott, O.; Olson, A. J. AutoDock Vina: Improving the speed and accuracy of docking with a new scoring function, efficient optimization, and multithreading. *J Comput Chem* **2010**, *31* (2), 455-461.
(14) Bartual, S. G.; Straume, D.; Stamsås, G. A.; Muñoz, I. G.; Alfonso, C.; Martínez-Ripoll, M.; Håvarstein, L. S.; Hermoso, J. A. Structural basis of PcsB-mediated cell separation in Streptococcus pneumoniae. *Nat Commun* **2014**, *5* (1).
